# Supplementary material for: Early mucosal responses following a randomised controlled human inhaled infection with attenuated Mycobacterium bovis BCG
Source: Nat Commun. 2025 May 29;16:4989. doi: 10.1038/s41467-025-60285-4 (PMC12122720; doi:10.1038/s41467-025-60285-4)
Supplement: Supplementary file 1 — Supplementary Information [file 41467_2025_60285_MOESM1_ESM.pdf]

Supplementary information

Contents:

- Supplementary Figure 1: Solicited adverse event frequency between BCG and saline volunteers
- Supplementary Figure 2: Immunohistochemistry staining of endobronchial biopsies
- Supplementary Figure 3: Mycobacterial Growth Inhibition Assay
- Supplementary Figure 4: Assigning cell type to clusters
- Supplementary Table 1: Volunteer information (1A volunteer visits; 1B Baseline characteristics by group)
- Supplementary Table 2: Immunohistochemistry staining methods
- Supplementary Note 1: Additional clinical results
- Supplementary Note 2: Antibodies used in flow cytometry panels and gating strategies

Supplementary Figure 1: Solicited adverse event frequency between BCG and saline volunteers

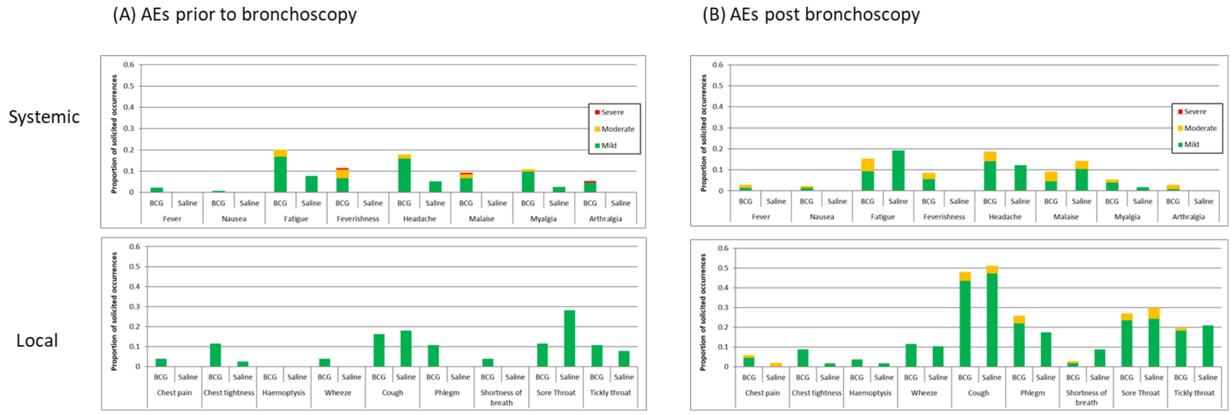

Frequency of Adverse Events (AEs) reported on two week volunteer electronic diary following BCG Danish 1 x 10^7 CFU or saline inhalation and **(A)** prior to bronchoscopy or **(B)** following bronchoscopy. Percent of systemic or local (respiratory) adverse events (AEs) by symptom, out of total solicited occurrences. AEs calculated as percent of total solicited occurrences. AEs were collected every 12 hours for 2 days following challenge then daily. n=20 BCG and n= 6 Saline. Bronchoscopy occurred at day 2 (D2; Group 1) or D7 (Group 2) following aerosol infection. **(A)** AEs shown only prior to bronchoscopy (Group 1 volunteers 2 days of AEs, Group 2 volunteers 7 days of AEs). Total solicited occurrences calculated as number of volunteers x 6.5 time points (average time points between groups: 4 time points for D2 volunteers and 9 time points for D7 volunteers, even numbers per group). **(B)** Bronchoscopy occurred D2 (Group 1) or D7 (Group 2) following BCG SSI AJV infection. Solicited occurrences calculated as number of volunteers x 9.5 time points (average time points between groups: 12 time points for D2 volunteers and 7 time points for D7 volunteers, even numbers per group).

Supplementary Figure 2: Immunohistochemistry staining of endobronchial biopsies

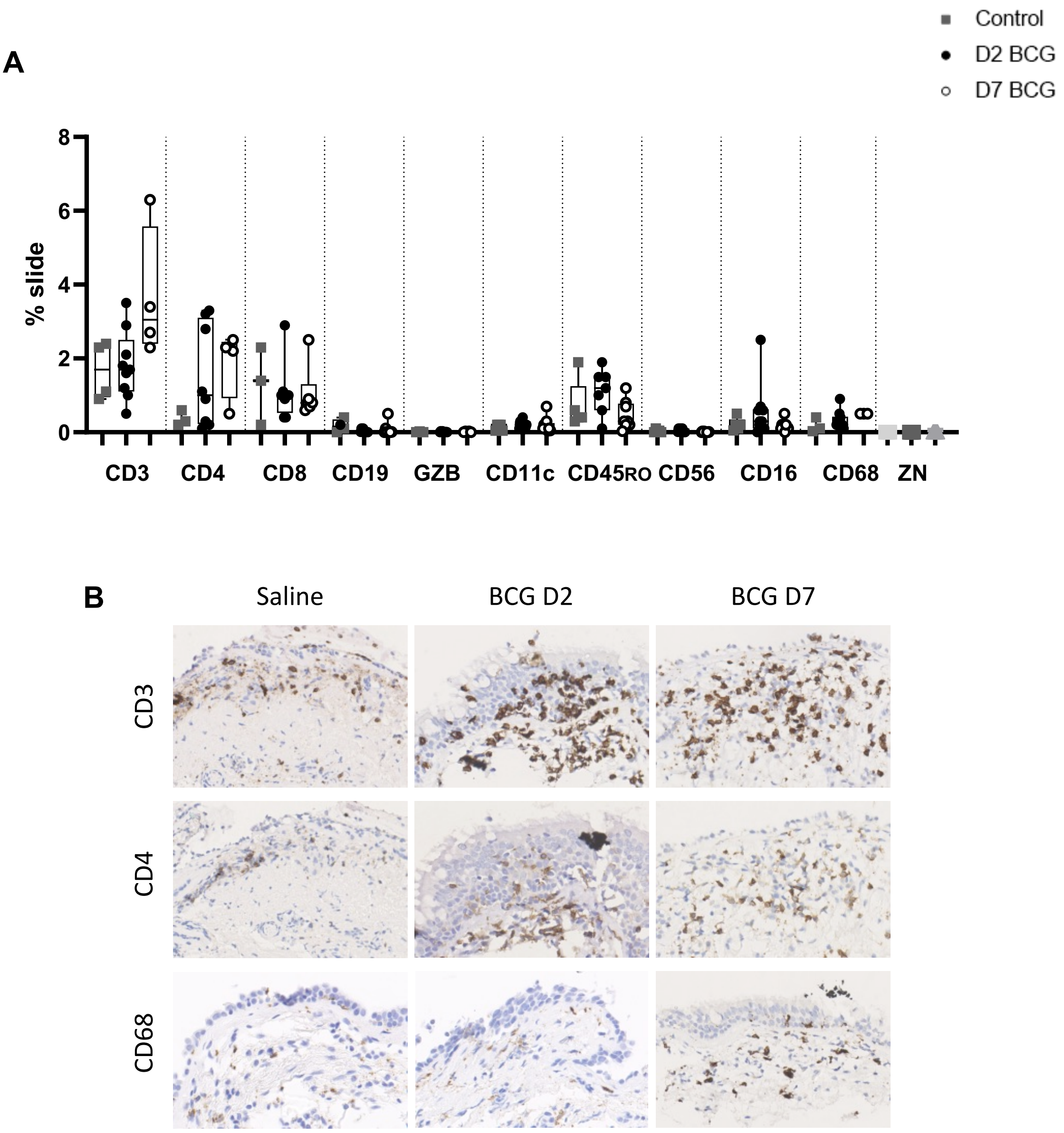

Immunohistochemistry staining of endobronchial biopsies. Endobronchial biopsies were collected at 2 days (D2) or 7 days (D7) following either saline (control) or BCG Danish inhalation, fixed, sectioned and stained. Digital slides were evaluated with image analysis software Nikon NIS-Ar to calculate the area of the tissue section (% slide) positively stained against the different cell markers **(A)**, using appropriate thresholds for each marker and Regions of Interest**.** ZN: Ziehl-Neelsen stain (for BCG detection) **(B)** is a representative digital image of sections from saline controls, D2 or D7 post BCG infection stained for CD3, CD4 or CD68. IHC stain (brown); Hematoxylin (nuclei blue) and eosin (extracellular matrix & cytoplasm pink) stain.

Supplementary Figure 3: Mycobacterial Growth Inhibition Assay post-BCG or saline inhalation

A

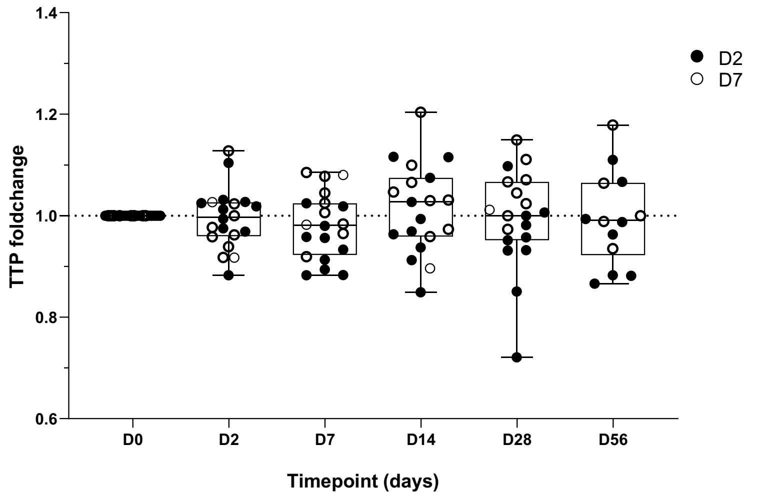

B

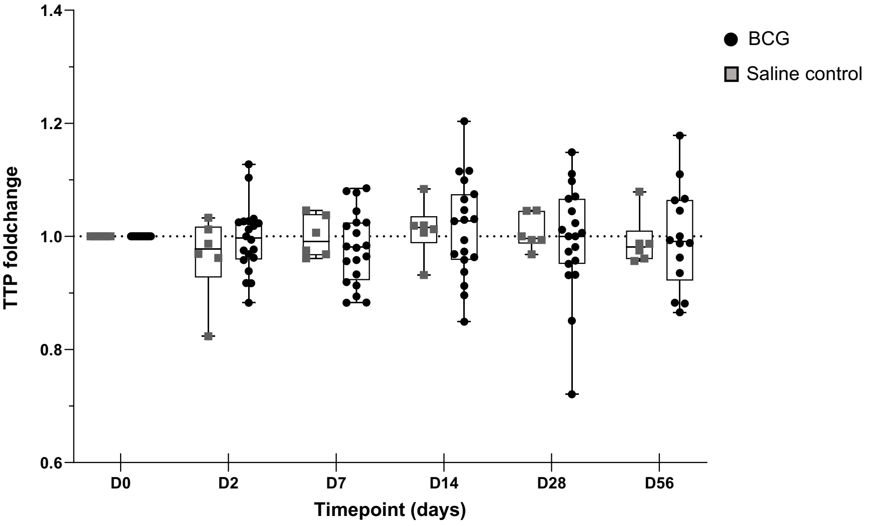

Mycobacterial Growth Inhibition Assay, of BCG-infected volunteers only (A) or saline vs BCG-infected volunteers (B) using PBMC and autologous serum. Time-To-Positivity (TTP) fold change calculated as TTP/TTP baseline. A higher TTP represents better mycobacterial growth control. A: Solid dots indicate Group 1 (D2 bronchoscopy) volunteers and circles represent Group 2 (D7 bronchoscopy) volunteers. B: Grey squares indicate saline controls and black dots indicate BCG-infected volunteers. Box and whisker plots show median, IQR and min/max. No difference between groups

Supplementary Figure 4: Assigning cell type to clusters

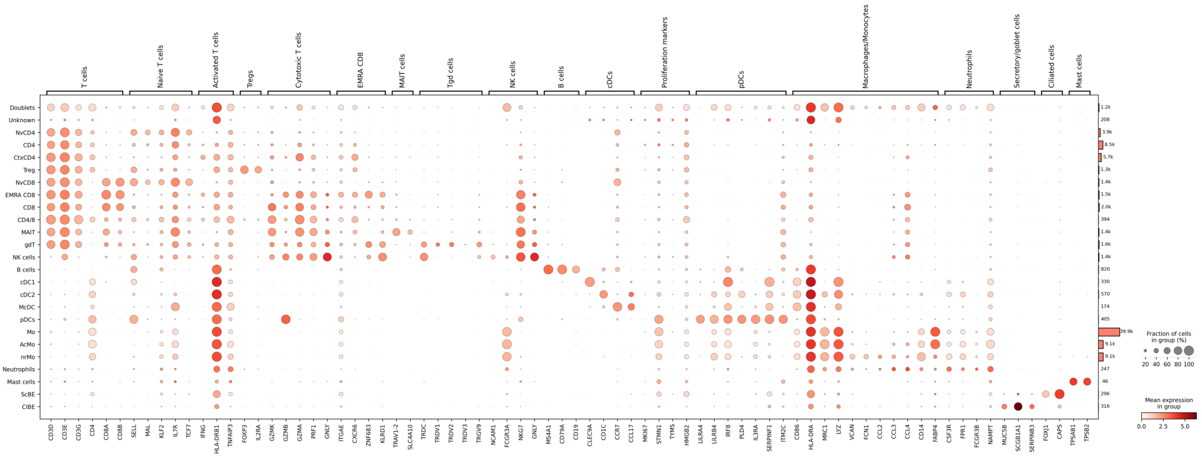

Markers used to identify each cell type:

Neutrophils [1-3]: *FCGR3B* (CD16b), *CSF3R* (Colony stimulating factor 3), *NAMPT* (Nicotinamide phosphoribosyltransferase), FPR1 (formyl peptide receptor 1). **Mo (Macrophages)** [4, 5] [3, 6, 7]: *MRC1* (mannose receptor/CD206), *LYZ* (Lysozyme). **nrMo (non-resident monocyte/macrophage)**: *VCAN* (versican), *FCN1* (Ficolin 1), *CCL2* (C-C Motif Chemokine Ligand 2). **AcMϕ (Activated macrophage)**: *CCL3* (C-C Motif Chemokine Ligand 3), *CCL4* (C-C Motif Chemokine Ligand 4). **NK cells**: *NCAM1* (CD56), *FCGR3A* (CD16), *CD3G* (CD3 negative). **Plasmacytoid dendritic cells (pDCs)** [3, 8, 9]: *IL3RA* (Interleukin 3 Receptor Subunit Alpha), *GZMB* (Granzme B), *SERPINF1*: Serpin Family F Member 1), *ITM2C* (Integral Membrane Protein 2C). **Conventional dendritic cells** (cDC) [8-12]: **cDC1**: *CLEC9A* (C-Type Lectin Domain Containing 9A). **cDC2**: *CD1C*. **Migratory cDC (McDC):** *CCR7* (C-C Motif Chemokine Receptor 7), *CCL17* (C-C Motif Chemokine Ligand 17). **γδ T cells**: *TRDC* (TCR delta constant), *TRDV2* (T cell receptor variable 2), *TRGV9* (T cell receptor gamma variable 9). **MAIT cells** [13]: *TRAV1-2* (Vα7.2), *SLC4A10* (Solute Carrier Family 4 Member 10). **T cells**: *CD3G*, *CD4*, *CD8A*, *CD8B*. **Tregs**:, *FOXP3* (Forkhead Box P3), *IL2RA* (Interleukin 2 Receptor Subunit Alpha). **Naïve marker**: *SELL* (Selectin L), *CCR7*, *KLF2* (KLF Transcription Factor 2), *MAL* (Mal, T Cell Differentiation Protein), *IL7R* (Interleukin 7 Receptor). **EMRA CD8 (terminally differentiated effector memory cells re-expressing CD45RA like CD8+ T cells)**: *ZNF683* (Zinc Finger Protein 683), *ITGAE* (Integrin Subunit Alpha E), *CXCR6* (C-X-C Motif Chemokine Receptor 6). CtxCD4 (Cytotoxic-like CD4+ T cells): *GZMA* (Granzyme A). **Bronchial epithelial cells**: **Ciliated bronchial epithelial cells(CIBE)**: *FOXJ1* (Forkhead Box J1) and *CAPS* (Calcyphosine). **Secretory and/or goblet cells (ScBE)**: *MUC5AC* (Mucin 5AC antibodies), *SCGB1A1* (Secretoglobin Family 1A Member 1) and SERPINB3 (Serpin Family B Member 3). **B cells**: *CD79A* (part of B cell receptor), *MS4A1* (CD20).
Gene abbreviations taken from the Genecard database [12]. Marker gene expression of cell types. The dot size represents the percentage of cells expressing the gene in the cell type. The dot colour represents the average expression of the gene in the cell type. The bar chart on the right shows the number of cells in each cell type.

Supplementary Table 1: Volunteer information

1A: Volunteer visits

Group	Vol no	D0 Challenge	D2	**D7 Bronch**	D14	D28	D56	D84	D168
2A	TBT-04301024	11/09/2019	13/09/2019	**18/09/2019**	26/09/2019	09/10/2019	06/11/2019	03/12/2019	26/02/2020
2A	TBT-04301026	23/10/2019	25/10/2019	**30/10/2019**	06/11/2019	20/11/2019	18/12/2019	14/01/2020	26/03/2020
2A	TBT-04301027	09/10/2019	11/10/2019	**16/10/2019**	23/10/2019	06/11/2019	04/12/2019	02/01/2019	26/03/2020
2A	TBT-04301028	20/11/2019	22/11/2019	**28/11/2019**	10/12/2019	19/12/2019	~~14/01/2020~~	13/02/2020	07/05/2020
2A	TBT-04301029	23/10/2019	25/10/2019	**30/10/2019**	06/11/2019	20/11/2019	02/01/2020	13/01/2019	30/03/2020
2A	TBT-04301030	23/10/2019	25/10/2019	**30/10/2019**	06/11/2019	20/11/2019	18/12/2019	14/01/2019	27/03/2020
2A	TBT-04301033	20/11/2019	22/11/2019	**28/11/2019**	09/12/2019	18/12/2019	14/01/2019	18/02/2020	07/05/2020
2A	TBT-04301031	21/01/2020	23/01/2020	**29/01/2020**	04/02/2020	18/02/2020	30/03/2020	21/04/2020	30/07/2020
2A	TBT-04301037	21/01/2020	23/01/2020	**29/01/2020**	04/02/2020	18/02/2020	04/03/2020	29/04/2020	16/07/2020
2A	TBT-04301042	12/02/2020	14/02/2020	**19/02/2020**	26/02/2020	11/03/2020	07/04/2020	06/05/2020	16/07/2020
2B	TBT-04301025	15/01/2020	17/01/2020	**22/01/2020**	29/01/2020	13/02/2020	11/03/2020	27/03/2020	16/07/2020
2B	TBT-04301034	15/01/2020	17/01/2020	**22/01/2020**	29/01/2020	12/02/2020	11/03/2020	24/03/2020	16/07/2020
2B	TBT-04301038	15/01/2020	17/01/2020	**22/01/2020**	29/01/2020	12/02/2020	11/03/2020	24/03/2020	16/07/2020

	Completed
	Telephone visit instead
	Visit but no induced sputum
	Visit out of window (one day late)
~~Missed~~	Missed visit

All Group 1 volunteer were completed as per protocol and were within window. Volunteer TBT-04301028's Day 56 visit was missed due to unforeseen volunteer travel. Volunteer TBT-04301029 D56 was one day of out window but was included in analysis as this was perceived as unlikely to significantly impact results given the window range was 28 days. All other changed visits were due to the lockdown of university facilities during the 2020 COVID-19 pandemic. TB043 was paused to all face-to-face visits from 23rd March through to the beginning of July 2020, to comply with UK government policy and regulatory advice. Adverse event (AE) data was recorded via telephone at these time points, and blood was not collected. When follow up visits recommenced, induced sputum procedures were not performed, to reduce the risk of spread of SARS-CoV-2 virus due to aerosol generating procedures. Group A are volunteers who inhaled BCG and Group B are volunteers who inhaled saline.

1B: Baseline characteristics by group

Characteristic		BCG Aerosol (n=20)		Saline Aerosol (n=6)	
		**Group 1A (bronchoscopy D2)**	**Group 2A (bronchoscopy D7)**	**Group 1B (bronchoscopy D2)**	**Group 2B (bronchoscopy D7)**
Female, n (%)		8 (80%)	7 (70%)	3 (100%)	1 (33%)
Median age, years (IQR)		27.8 (22.5-35.9)	23.4 (20.4-30.9)	29.1 (28.0-31.7)	22.2 (19.0-31.3)
Median BMI, kg/m^2 (IQR)		23.0 (20.2-29.2)	23.9 (21.1-25.8)	25.4 (21.5-28.2)	24.5 (23.7-25.3)
Lung function					
Median % predicted FEV_1/FVC (IQR)		97.1 (95.7-98.6)	98.6 (94.9-100.3)	93.5 (89.9-98.8)	100.0 (90.0-105.0)
Median % predicted TLCO (range)		107.1 (99.8-117.2)	96.6 (91.6-105.8)	108.5 (100.0-122.9)	110.0 (95.0-117.0)
Median baseline % SaO2 (IQR)		98.0 (97.0-99.0)	98.0 (97.8-98.3)	98.0 (97.0-98.0)	98.0 (98.0-99.0)
Country of birth, n (%)	UK	6 (60%	10 (100%)	2 (67%)	3 (100%)
	Australia	2 (20%			
	Germany			1 (33%)	
	Canada	2 (20%)			
Self-identified ethnicity, n (%)	Caucasian	8 (80%)	10 (100%)	3 (100%)	3 (100%)
	Chinese	1 (10%)			
	Mixed heritage	1 (10%)			

Supplementary Table 2: Immunohistochemistry staining methods

Reagent	Supplier	Catalogue No.	Antigen retrieval	Primary antibody concentration
CD3	Leica Biosystems	LN10	Heat induced antigen retrieval 20 minutes using Leica ER2 (high pH).	Diluted 1 in 100
CD4	Leica Biosystems	4B12	Heat induced antigen retrieval 20 minutes using Leica ER2 (high pH).	Antibody used at stock concentration
CD8	Dako	C8/144B	Heat induced antigen retrieval 20 minutes using Leica ER2 (high pH).	Diluted 1 in 80
CD11c	Leica Biosystems	PA0554	Heat induced antigen retrieval 20 minutes using Leica ER2 (high pH)	Antibody used at stock concentration
CD16	Leica Biosystems	NCL-L-CD16	Heat induced antigen retrieval 30 minutes using Leica ER1 (low pH).	1/80 of 18mg/L
CD56	Leica Biosystems	PA0191	Heat induced antigen retrieval 30 minutes using Leica ER1 (low pH)	Antibody used at stock concentration
CD19	Leica Biosystems	BT51E	Heat induced antigen retrieval 20 minutes using Leica ER2 (high pH)	Antibody used at stock concentration
CD45RO	Leica Biosystems	PA0146	Heat induced antigen retrieval 5 minutes using Leica ER1 (low pH)	Antibody used at stock concentration
GRANB	Leica Biosystems	11F1	Heat induced antigen retrieval 20 minutes using Leica ER2 (high pH)	Antibody used at stock concentration
CD68	Dako	PG-M1	Heat induced antigen retrieval 20 minutes using Leica ER2 (high pH).	Diluted 1 in 200

Supplementary Note 1: Additional clinical results

There was no significant difference in frequency of total AEs, or respiratory or systemic AEs between volunteers that received BCG and those that received saline, either prior to or following bronchoscopy. However, following aerosol BCG inhalation some volunteers reported transient fever (n=3/20, 15%), feverishness (n=8/20, 40%), malaise (n=7/20, 35%), arthralgia (n=4/20, 20%), chest pain (n=3/20, 15%), shortness of breath (n=3/20, 15%) and wheeze (n=2/20, 10%), and these symptoms were not experienced by volunteers who inhaled saline. All AEs reported post bronchoscopy were consistent with expected bronchoscopy related AEs and all were self-limiting. However, post-bronchoscopy, five (25%) BCG recipients experienced arthralgia, ten (50%) reported feverishness and three (15%) arthralgia or fever, which were not reported in control volunteers.

There was no difference in the incidence of severe AEs reported either post-aerosol or post-bronchoscopy between BCG and saline inhalation volunteers. However, amongst those volunteers who did report an AE post-bronchoscopy, the AE was more likely to be moderate than mild in BCG compared with saline volunteers (Chi square, fisher's exact p=0.008).

There was no significant change in spirometry following either BCG or saline inhalation (FEV_1 median reduction in BCG group -32.7 (IQR -61.3 to 8.6); saline group -25.0 (IQR-35.4 to 27.6), p=0.53); FVC median reduction in BCG group -11.9 (IQR -69.3 to 19.8); saline group –19.8 (IQR -35.2 to 14.0), p=0.98 on Mann Whitney). There was also no significant difference between BCG and saline recipients at their D7 TLCO, nor any difference in D7 TLCO between the BCG volunteers who had already a bronchoscopy (Group 1A, D2 bronchoscopy) and BCG volunteers prior to bronchoscopy (Group 2A, D7 bronchoscopy).

Related unsolicited AEs were clinically unremarkable and there were no possibly or probably related grade 3 unsolicited AEs. There were no recurring patterns of unsolicited AEs that were probably or definitely related to BCG aerosol infection.

Supplementary Note 2: Flow cytometry: BAL sample size and counts, antibody panels and gating strategies

BAL cells were used sequentially from Panel 1 to Panel 4 depending on sample availability. All cells were stained with the innate cell panel (Panel 1) which was the primary objective of the study. Cells from blood were stained for all panels, with some missing samples at later timepoints (D56 onwards) in Group 2 due to restrictions on visits due to the COVID-19 pandemic as outlined in Supplementary Figure 1A.

Sample Size

BAL samples	D2 saline	D2 BCG	D7 saline	D7 BCG
**Panel 1: Innate cells**	100% (3/3)	100% (10/10)	100% (3/3)	100% (10/10)
**Panel 2: T cells, γδ T cells**	100% (3/3)	80% (8/10)	100% (3/3)	90% (9/10)
**Panel 2 with cytokine stimulation (IFN-γ)**	67% (2/3)	30% (3/10)	100% (3/3)	70% (7/10)
**Panel 3: DURTs**	0	30% (3/10)	0	40% (4/10)
**Panel 4: Additional cytokines (IL-2, TNF-a, IL-17**	0	0	0	20% (2/10)-data not shown due to low sample size

Cell counts (singlets)

BAL samples	D2 saline	D2 BCG	D7 saline	D7 BCG
**Samples**	3	10	3	10
**Median**	$1.82x10^6$	$1.94\ x10^6$	$6.07\ x10^5$	$6.85\ x10^5$
**IQR**	$1.1\text{-}2.9\ x10^6$	$1.1\text{-}3.4\ x10^6$	$6.0\text{-}6.8\ x10^5$	$4.2\text{-}9.7\ x10^5$

**PANEL 1**<br><br>**BAL or Whole blood**	**Granulocytes, Natural Killer cells, Antigen presenting cells, CD3+CD56+ cells, HLA-DR+ T cells**	
**Marker**	**Colour**	**Supplier**
Live/Dead	Red	Invitrogen
CD19	ECD	Biolegend
CD11c	BV785	Biolegend
CD14	BV421	ebioscience

CD16	AF488	Biolegend
CD206	APC-CY7	Biolegend
CD3	BUV496	BD Biosciences
CD326	PercpCy5.5	Biolegend
CD45	BV650	Biolegend
CD56	PECY5	Invitrogen
CD66b	AF700	Biolegend
CD86	APC	Biolegend
HLA-DR	PE	Biolegend
Siglec8	PE-Cy7	Biolegend
**PANEL 2**<br>**BAL or Whole blood ICS**	**CD4+ T cells, CD8+ T cells, γδ T cells, Antigen-specific IFN-γ+ detection**	
**Marker**	**Colour**	**Supplier**
Live/Dead	Red	Invitrogen
CD19	ECD	Biolegend
CD103	PE	Biolegend
CD14	PE dazzle	Biolegend
CD153	AF647	R&D Systems
CD29	APC-Cy7	Biolegend
CD4	Pacific Blue	Biolegend
CD49D	BV605	Biolegend
CD56	BV785	Biolegend
CXCR3	PerCpCy5.5	Biolegend
KLRG-1	AF488	Biolegend
PD-1	BUV395	BD Biosciences
γδ pan:<br>B1 (11F2)	BV711	BD Biosciences
γδ2: (B6)	BV480	BD Biosciences
CD3*	AF700	ebioscience

CD8a*	BV650	Biolegend
IFN-y*	PE-CY7	Life Technologies
**Panel 3**<br>**BAL or PBMCs**	**MAIT cells, iNKT cells, including CD4 CD8 subsets**	
**Marker**	**Colour**	**Supplier**
Live/Dead	Aqua	Invitrogen
MR1 tetramer:<br>5-OP-RU	APC	NIH Tetramer Core facility
MR1 tetramer control:<br>6-FP	AF488	NIH Tetramer Core facility
CD14	PerCpCy5.5	Biolegend
CD161	BV785	Biolegend
CD19	BV510	BD Biosciences
CD26	PE	Biolegend
CD27	BV605	Biolegend
CD3	AF700	ebioscience
CD4	BUV395	BD Biosciences
CD8a	APC-H7	BD Biosciences
HLADR	BV650	Biolegend
Vα24	PercpCy5.5	Biolegend
Vα7.2	BV421	Biolegend
Vβ 11	PE-vio770	Miltenyi
**Panel 4**<br>**BAL or Whole blood ICS**	**Antigen specific cytokine production by monocytes and T cells**	
**Marker**	**Colour**	**Supplier**
Live/Dead	Aqua	Invitrogen
CD14	PerCpCy5.5	Biolegend
CD16	APC-Cy7	Biolegend

CD19	BV510	BD Biosciences
CD4	Pacific Blue	Biolegend
CD3*	AF700	ebioscience
CD8a*	BV650	Biolegend
IFN-y*	PE-CY7	Life Technologies
IL-17*	AF488	Biolegend
IL-2*	PE	Beckman coulter
TNF-α*	AF647	Biolegend

* Intracellular staining post-permeabilisation. (For BAL cells, if there were insufficient cells for antigen specific response detection, cells were only surface stained for Panel 2 by adding CD3 and CD8a to the surface stain).

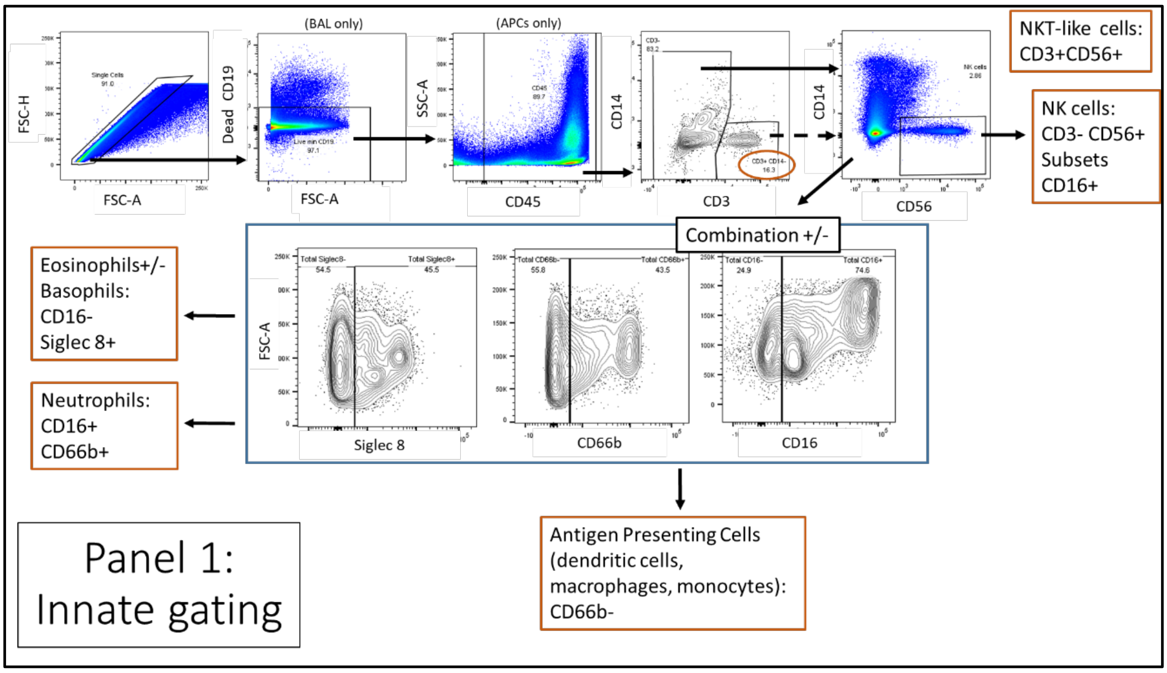

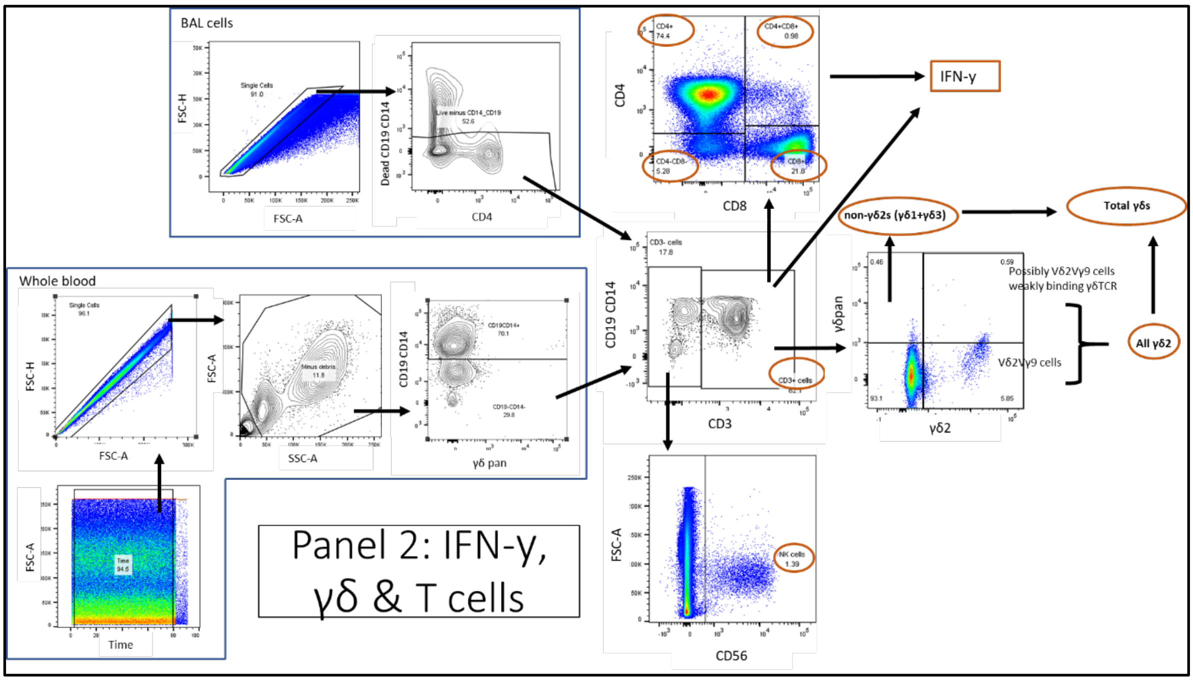
BAL cells
Whole blood
FSC-H
FSC-A
Single Cells
Dead CD19 CD14
CD4
Live minus CD14_CD19
SSC-A
Minus debris
CD19 CD14
γδ pan
CD19CD14+
CD19-CD14-
Time
CD3- cells
CD3+ cells
CD3
CD8
CD4+
74.4
CD4+CD8+
0.98
CD4-CD8-
5.28
CD8+
21.8
IFN-γ
non-γδ2s (γδ1+γδ3)
Total γδs
Possibly Vδ2Vγ9 cells
weakly binding γδTCR
Vδ2Vγ9 cells
All γδ2
γδ2
CD56
NK cells
1.39
Panel 2: IFN-γ,
γδ & T cells

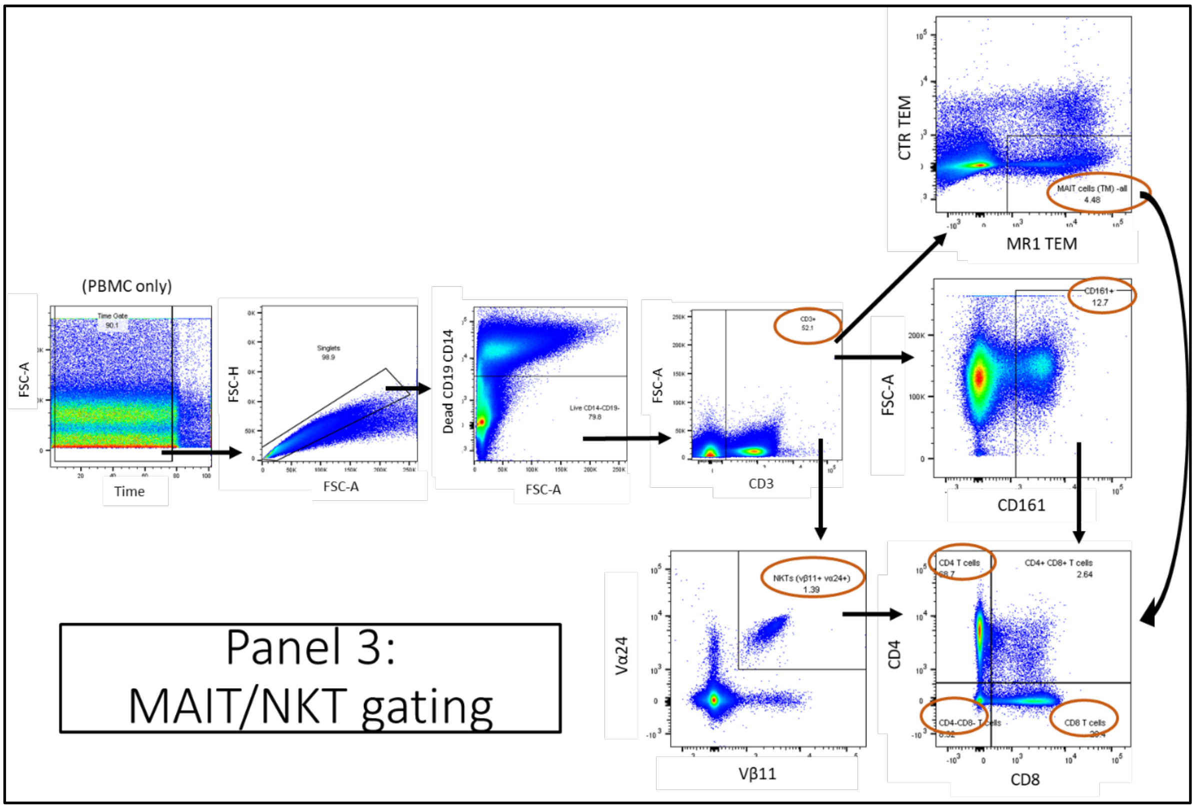
(PBMC only)
FSC-A
Time
FSC-H
Singlets
98.9
Dead CD19 CD14
Live CD14-CD19-
79.8
CD3+
52.1
CD3
CTR TEM
MR1 TEM
MAIT cells (TM) -all
4.48
CD161
CD161+
12.7
Vα24
Vβ11
NKTs (vβ11+ vα24+)
1.39
CD4
CD8
CD4 T cells
CD4+ CD8+ T cells
2.64
CD4-CD8- T cells
CD8 T cells
Panel 3:
MAIT/NKT gating

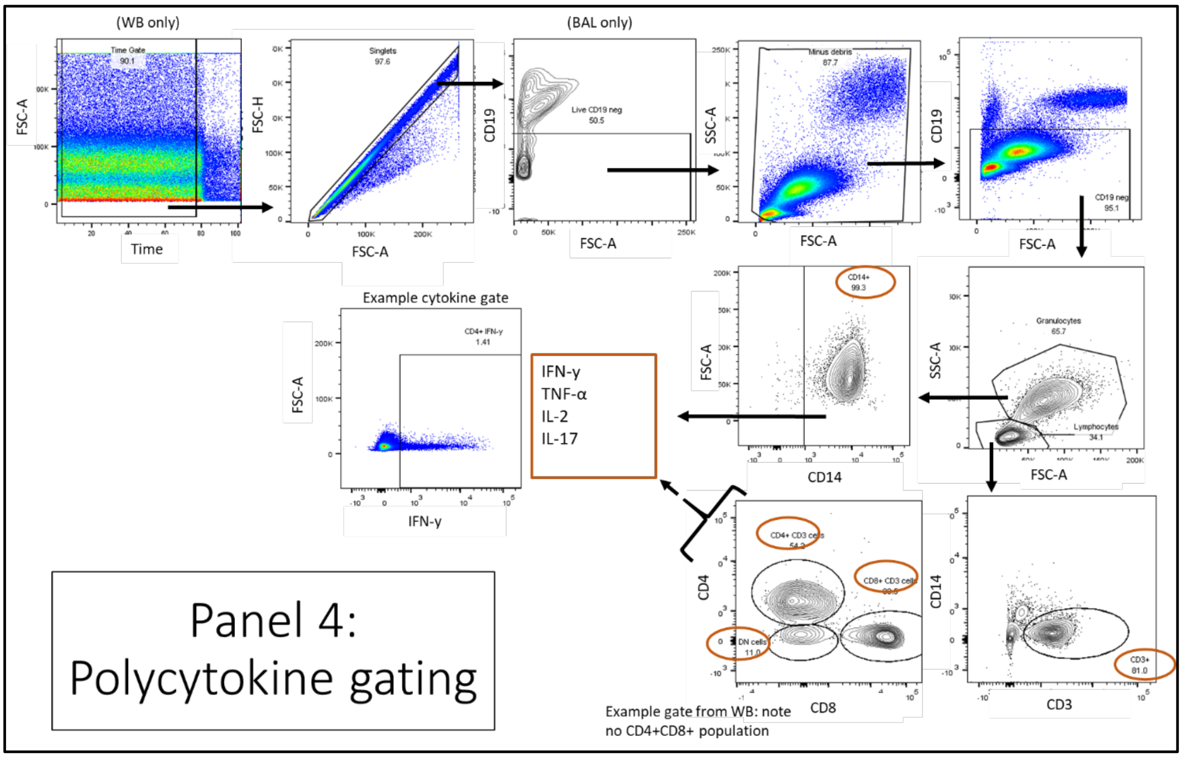
(WB only)
(BAL only)
Time Gate 90.1
Singlets 97.6
Live CD19 neg 50.5
Minus debris 87.7
CD19 neg 95.1
FSC-A
FSC-H
CD19
SSC-A
Time
Example cytokine gate
CD4+ IFN-γ 1.41
IFN-γ
TNF-α
IL-2
IL-17
CD14+ 99.3
CD14
Granulocytes 65.7
Lymphocytes 34.1
CD4+ CD3 cells 54.2
CD8+ CD3 cells
DN cells 11.0
CD3+ 81.0
CD4
CD8
CD3
Example gate from WB: note no CD4+CD8+ population
Panel 4: Polycytokine gating

References

1. FitzGerald ES, Jamieson AM. Unique transcriptional changes in coagulation cascade genes in SARS-CoV-2-infected lung epithelial cells: A potential factor in COVID-19 coagulopathies. bioRxiv : the preprint server for biology. 2020:2020.07.06.182972. doi: 10.1101/2020.07.06.182972. PubMed PMID: 32676594.
2. Shaath H, Vishnubalaji R, Elkord E, Alajez NM. Single-Cell Transcriptome Analysis Highlights a Role for Neutrophils and Inflammatory Macrophages in the Pathogenesis of Severe COVID-19. Cells. 2020;9(11):2374. PubMed PMID: doi:10.3390/cells9112374.
3. Chua RL, Lukassen S, Trump S, Hennig BP, Wendisch D, Pott F, et al. COVID-19 severity correlates with airway epithelium–immune cell interactions identified by single-cell analysis. Nature Biotechnology. 2020;38(8):970-9. doi: 10.1038/s41587-020-0602-4.
4. Gren ST, Rasmussen TB, Janciauskiene S, Håkansson K, Gerwien JG, Grip O. A Single-Cell Gene-Expression Profile Reveals Inter-Cellular Heterogeneity within Human Monocyte Subsets. PLOS ONE. 2015;10(12):e0144351. doi: 10.1371/journal.pone.0144351.
5. Wong KL, Tai JJ-Y, Wong W-C, Han H, Sem X, Yeap W-H, et al. Gene expression profiling reveals the defining features of the classical, intermediate, and nonclassical human monocyte subsets. Blood. 2011;118(5):e16-e31. doi: 10.1182/blood-2010-12-326355.
6. Liao M, Liu Y, Yuan J, Wen Y, Xu G, Zhao J, et al. Single-cell landscape of bronchoalveolar immune cells in patients with COVID-19. Nature Medicine. 2020;26(6):842-4. doi: 10.1038/s41591-020-0901-9.
7. Morse C, Tabib T, Sembrat J, Buschur K, Bittar HT, Valenzi E, et al. Proliferating SPP1/MERTK-expressing macrophages in idiopathic pulmonary fibrosis. European Respiratory Journal. 2019:1802441. doi: 10.1183/13993003.02441-2018.
8. Villani A-C, Satija R, Reynolds G, Sarkizova S, Shekhar K, Fletcher J, et al. Single-cell RNA-seq reveals new types of human blood dendritic cells, monocytes, and progenitors. Science. 2017;356(6335):eaah4573. doi: 10.1126/science.aah4573.
9. Franzén O, Gan L-M, Björkegren JLM. PanglaoDB: a web server for exploration of mouse and human single-cell RNA sequencing data. Database. 2019;2019. doi: 10.1093/database/baz046.
10. Kedl RM, Lindsay RS, Finlon JM, Lucas ED, Friedman RS, Tamburini BAJ. Migratory dendritic cells acquire and present lymphatic endothelial cell-archived antigens during lymph node contraction. Nature Communications. 2017;8(1):2034. doi: 10.1038/s41467-017-02247-z.
11. Alferink J, Lieberam I, Reindl W, Behrens A, Weiß S, Hüser N, et al. Compartmentalized Production of CCL17 In Vivo : Strong Inducibility in Peripheral Dendritic Cells Contrasts Selective Absence from the Spleen. Journal of Experimental Medicine. 2003;197(5):585-99. doi: 10.1084/jem.20021859.
12. Fishilevich S, Nudel R, Rappaport N, Hadar R, Plaschkes I, Iny Stein T, et al. GeneHancer: genome-wide integration of enhancers and target genes in GeneCards. Database (Oxford). 2017;2017. Epub 2017/06/13. doi: 10.1093/database/bax028. PubMed PMID: 28605766; PubMed Central PMCID: PMCPMC5467550.
13. Huang H, Sikora MJ, Islam S, Chowdhury RR, Chien Y-h, Scriba TJ, et al. Select sequencing of clonally expanded CD8⁺ T cells reveals limits to clonal expansion. Proceedings of the National Academy of Sciences. 2019;116(18):8995. doi: 10.1073/pnas.1902649116.
14. Zhou Y, Zhou B, Pache L, Chang M, Khodabakhshi AH, Tanaseichuk O, et al. Metascape provides a biologist-oriented resource for the analysis of systems-level datasets. Nat Commun. 2019;10(1):1523. Epub 2019/04/05. doi: 10.1038/s41467-019-09234-6. PubMed PMID: 30944313; PubMed Central PMCID: PMCPMC6447622.

CLINICAL TRIAL PROTOCOL

A human challenge study to evaluate innate and adaptive immune responses to a controlled human infection with BCG administered by the aerosol inhaled route in healthy, BCG-naïve, UK adult volunteers

Short title: Investigating immune responses to aerosol BCG challenge in healthy UK adults

**Trial Reference**:	TB043
**REC Reference:**	
**IRAS Reference:**	
**Date and Version Number**	24 April 2018, v1.0
**Chief Investigator:**	Professor Helen McShane
**Sponsor:**	University of Oxford
**Safety Monitoring Committee:**	Dr Hassan Mahomed
	Professor Stephen Gordon
	Francesca Little
**Funding body:**	The Wellcome Trust
**Author:**	Dr Julia Marshall and Dr Rebecca Powell Doherty

Confidentiality Statement

This document contains confidential information that must not be disclosed to anyone other than the Sponsor, the Investigator Team, and members of the Research Ethics Committee, unless authorised to do so. This information cannot be used for any purpose other than the evaluation or conduct of the clinical investigation without the prior written consent of Professor Helen McShane.

TABLE OF CONTENTS

1 STATEMENT OF COMPLIANCE 5

2 KEY STUDY CONTACTS AND ROLES 6

3 SYNOPSIS 8

3.1 Synopsis 8

3.2 Schedule of visits and procedures 10

4 ABBREVIATIONS 11

5 BACKGROUND AND RATIONALE 12

5.1 Context 12

Challenge models 13

Mycobacterial growth inhibition assay and correlates of protection 13

Basis for the aerosol inhaled route 14

Aerosol delivery devices 14

TB041: A clinical challenge trial to evaluate controlled human infection with BCG administered by the aerosol-inhaled route compared with the intradermal route in healthy, BCG-naïve, UK adult volunteers. Clinicaltrials.gov NCT02709278 15

Bronchoscopy and bronchoalveolar sampling 15

5.2 Rationale 16

Hypothesis 16

BCG and dosage 16

5.3 Risks and Benefits 18

Potential risks 18

Potential benefits 20

6 OBJECTIVES AND OUTCOME MEASURES 20

Primary Objective 20

7 PARTICIPANT IDENTIFICATION 21

7.1 Study Participants 21

8 STUDY DESIGN AND PROCEDURES 23

8.1 Study Numbers and Groups 23

8.2 Recruitment 23

8.3 Informed Consent 24

8.4 Screening and Eligibility Assessment 25

8.5 Randomisation and blinding 25

8.6 Follow up visits 25
8.7 Timepoints 26
8.8 Sample handling 26
8.9 Challenge Postponement Criteria 27
8.10 Bronchoscopy Postponement criteria 27
8.11 Discontinuation / Withdrawal Criteria 27
8.12 Safety 28
8.13 End of Study Definition 28
9 STUDY AGENTS AND DEVICES 28
9.1 BCG Description 28
BCG Bulgaria 28
Storage of BCG 28
9.2 Dispensing and administration 29
9.3 Saline 29
9.4 Salbutamol 29
9.5 MicroAIR NE-U22 29
9.6 Ultrasonic nebuliser NE-U780 29
9.7 Sedative & anaesthetic agents for bronchoscopy 29
10 ASSESSMENT OF SAFETY 30
10.1 Definitions 30
10.2 Foreseeable Adverse Reactions 31
10.3 Expected Serious Adverse Events 31
10.4 Causality Assessment 31
10.5 Reporting Procedures for All Adverse Events 32
10.6 Assessment of Severity 33
10.7 Procedures to be followed in the event of abnormal findings 34
10.8 Safety Monitoring Committee 34
11 STATISTICS 34
Primary objective 35
Secondary objective 35
Tertiary objective 35
12 DATA MANAGEMENT 36
12.1 Source Data 36
12.2 Access to Data 36
12.3 Data Recording and Record Keeping 36
13 QUALITY ASSURANCE PROCEDURES 36
13.1 Quality Assurance 36

13.2 Monitoring 37
14 SERIOUS BREACHES 37
15 ETHICAL AND REGULATORY CONSIDERATIONS 37
15.1 Declaration of Helsinki 37
15.2 Good Clinical Practice 37
15.3 Approvals 37
15.4 Reporting 38
15.5 Volunteer Confidentiality 38
15.6 Expenses and Benefits 38
16 FINANCE AND INSURANCE 38
16.1 Funding 38
16.2 Indemnity 38
16.3 Insurance 38
17 PUBLICATION POLICY 38
18 REFERENCES 39
19 Appendices: Appendix A 43

1 STATEMENT OF COMPLIANCE

Investigator Agreement

"I have read this protocol and agree to abide by all provisions set forth therein. I agree to comply with the International Conference on Harmonisation Tripartite Guideline on Good Clinical Practice."

Professor Helen McShane		24.04.2018
Chief Investigator	Investigator Signature	Date

Conflict of Interest

"According to the Declaration of Helsinki, 2008, I have read this protocol, and declare no conflict of interest with any Investigators"

Professor Helen McShane		24.04.2018
Chief Investigator	Investigator Signature	Date

2 KEY STUDY CONTACTS AND ROLES

**Clinical Trial Units:**	Centre for Clinical Vaccinology and Tropical Medicine (CCVTM)<br>Churchill Hospital,<br>Old Road<br>Headington<br>Oxford, OX3 7LE<br><br>Oxford University Hospital NHS Foundation Trust (OUH)<br>Headington<br>Oxford, OX3 7LE
**Chief Investigator:**	Professor Helen McShane<br>CCVTM<br>Tel: +44 (0)1865 617606<br>Email: helen.mcshane@ndm.ox.ac.uk
**Other Investigators:**	Dr Julia Marshall<br>CCVTM
**Lead Nurse:**	RN Raquel Lopez Ramon<br>CCVTM
**Collaborators:**	Dr Henry Bettinson<br>Consultant Respiratory and Intensive Care Physician<br>Oxford Centre for Respiratory Medicine<br>Oxford University Hospitals NHS Foundation Trust<br>Oxford, OX3 7LE<br>Tel: +44 (0)1865 225234<br>Email: henry.bettinson@ouh.nhs.uk<br><br>Dr Timothy Hinks<br>Senior Research Fellow and Honorary consultant Respiratory Medicine Unit<br>NDM Experimental Medicine<br>University of Oxford Rm 7406<br>Level 7, John Radcliffe Hospital<br>Oxford, OX3 9DU<br>Tel: +44 (0)1865 220885<br>Email: timothy.hinks@ndm.ox.ac.uk
**Project Managers:**	Dr Rebecca Powell Doherty & Dr Alison Lawrie<br>CCVTM
**Safety Monitoring Committee:**	Dr Hassan Mahomed<br>Division of Health Systems and Public Health<br>University of Stellenbosch<br>Francie van Zijl Drive<br>Tygerberg, 7505<br>South Africa<br>Tel: +27 21 938 9111

Email: Hassan.Mahomed@westerncape.gov.za

Professor Stephen Gordon
Director Malawi-Liverpool-Wellcome Trust Programme
Queen Elizabeth Central Hospital
College of Medicine
Chichiri
Malawi
Tel: +265 1874628
Email: sgordon@mlw.mw
stephen.gordon@lstmed.ac.uk

Francesca Little
SMC Statistician
PD Hahn Building, 5.36
Upper Campus
University of Cape Town
Tel: +27 21 650 3225
Email: francesca.little@uct.ac.za

Senior Immunologist:

Dr Iman Satti
The Jenner Institute
Old Road Campus Research Building
Roosevelt Drive, Headington
Oxford, OX3 7DQ
Tel: +44 (0)1865 617623
Fax: +44 (0)1865 617608
Email: iman.satti@ndm.ox.ac.uk

Statistician:

Nicola Williams
Senior Trial Statistician
Nuffield Department of Primary Care Health Sciences
Radcliffe Observatory Quarter, University of Oxford
Woodstock Road,
Oxford. OX2 6GG.
Tel: +44 (0) 1865 289300.
Email: Nicola.Williams@phc.ox.ac.uk

Sponsor:

Clinical Trials & Research Governance
Joint Research Office, University of Oxford
Boundary Brook House
Churchill Drive, Headington
Oxford, OX3 7LQ

Monitor:

Clinical Trials & Research Governance
Joint Research Office, University of Oxford
Boundary Brook House
Churchill Drive, Headington
Oxford, OX3 7LQ

3 SYNOPSIS

3.1 Synopsis

Trial Title	A human challenge study to evaluate innate and adaptive immune response to a controlled human infection with BCG administered by the aerosol inhaled route in healthy, BCG-naïve, UK adult volunteers	
Trial Identifier	TB043	
Chief Investigator	Professor Helen McShane	
Trial Centres	Centre for Clinical Vaccinology and Tropical Medicine (CCVTM), Churchill Hospital, Old Road, Headington, Oxford, OX3 7LE<br><br>Oxford University Hospital NHS Foundation Trust (OUH), Headington, Oxford, OX3 7LE	
Trial participants	Healthy adult volunteers aged 18-50 years	
Planned Sample Size	65 subjects<br>Group 1: 10 volunteers will receive 1 x 10^7 cfu aerosol inhaled BCG Bulgaria and 3 volunteers will receive aerosol inhaled normal saline placebo. All Group 1 volunteers will have a bronchoscopy 2 days post challenge<br>Group 2: 10 volunteers will receive 1 x 10^7 cfu aerosol inhaled BCG Bulgaria and 3 volunteers will receive aerosol inhaled normal saline placebo. All Group 2 volunteers will have a bronchoscopy 7 days post challenge<br>Group 3: 10 volunteers will receive 1 x 10^7 cfu aerosol inhaled BCG Bulgaria and 3 volunteers will receive aerosol inhaled normal saline placebo. All Group 3 volunteers will have a bronchoscopy 14 days post challenge<br>Group 4: 10 volunteers will receive 1 x 10^7 cfu aerosol inhaled BCG Bulgaria and 3 volunteers will receive aerosol inhaled normal saline placebo. All Group 4 volunteers will have a bronchoscopy 28 days post challenge<br>Group 5: 10 volunteers will receive 1 x 10^7 cfu aerosol inhaled BCG Bulgaria and 3 volunteers will receive aerosol inhaled normal saline placebo. All Group 5 volunteers will have a bronchoscopy 56 days post challenge	
Challenge Schedule	Single BCG challenge at day 0	
Follow-up Duration	24 weeks from challenge day	
Blood Sampling	See visit schedule	
Trial Interventions	Spirometry<br>BCG challenge by aerosol inhaled route<br>Saline placebo by aerosol inhaled route<br>Venepuncture<br>Bronchoscopy, BAL and endobronchial biopsy<br>Chest x-ray<br>Induced sputum	
Trial Duration	Estimated end date is June 2020	
Planned Trial Period	Planned start date is July 2018	
	Objectives	Outcome Measures
Primary	To define the systemic and mucosal innate and adaptive immune responses induced by aerosol inhaled BCG infection in healthy BCG and *M.tb* naïve UK adults	Laboratory markers of innate and adaptive immunity, including *ex-vivo* ELISpot and ELISAs; and RNA sequence analysis and intracellular cytokine staining or chip cytometry with blood, BAL and biopsy samples
Secondary	To identify laboratory markers of the immune response that correlate with the protection as defined by PBMC MGIA conducted at D56 post challenge	Established and exploratory markers of innate, cell mediated and humoral immunity in blood, BAL and biopsy samples; MGIA on PBMCs collected at Day 56

Tertiary	To describe the human clinical response to BCG challenge by the aerosol inhaled route in healthy, BCG-naïve UK adult volunteers	Actively and passively collected data on adverse events; detailed participant symptom profiles; Laboratory parameters including BCG CFU counts in induced sputum
Challenge agent	*Mycobacterium bovis* BCG	
Dose	1 x 10^7 cfu BCG Bulgaria	
Route of challenge	Aerosol inhalation by nebuliser	
Allocation Method	Sequential enrolment into groups (Groups 1-5), followed by within-group randomisation by sequentially numbered sealed envelopes (BCG or placebo)	

3.2 Schedule of visits and procedures

Table 1. Schedule of study procedures

**Visit number**	**1**	**2**	**3**	**4**	**5**	**6**	**7**	**8**	**9**
Timeline (days)	Screening	0	2	7	14	28	56	84	168
Timeline (weeks)	Screening	0		1	2	4	8	12	24
Time windows (days)			±1	±2	±5	±7	±14	±21	±28
Inclusion/exclusion criteria	X	X							
Review contra-indications	X	X	G1	G2	G3	G4	G5		
Informed consent	X	X	G1	G2	G3	G4	G5		
Medical history	X	(X)	(X)	(X)	(X)	(X)	(X)	(X)	(X)
Physical examination	X	(X)	(X)	(X)	(X)	(X)	(X)	(X)	(X)
Vital signs	X	X	X	X	X	X	X	X	X
Urinalysis	X								
PFTs	X	X	X	X	X	X			
β-HCG urine test (females)	X	X	G1	G2	G3	G4	G5		
**BCG challenge**		X							
**Bronchoscopy**			G1	G2	G3	G4	G5		
Chest radiograph	X								
Induced sputum*								X	X
Local & systemic events/reactions		X	X	X	X	X	X	X	X
E-diary #1 setup		X							
E-diary #1 final review					X				
7-day E-diary #2 for bronchoscopy symptoms					G3	G4	G5		
Biochemistry (mL)	4					4			
Haematology (mL)	2					2			
Coagulation (mL)	4								
HBV, HCV, HIV (mL)	10								
HLA typing (mL)		4							
Exploratory immunology +/- IGRA (mL)	10	60	60	60	60	60	60	60	60
Blood vol per visit (mL)	30	64	60	60	60	66	60	60	60
Cumulative blood vol (mL)	30	94	154	214	274	340	400	460	520

X Event scheduled to occur
(X) If considered necessary, emphasising any complaint or change in medications
G1-G5 Designates Group number
* No induced sputum for controls (Arm 2 volunteers)

4 ABBREVIATIONS

**AE**	Adverse Event
**AR**	Adverse Reaction
**BAL**	Bronchoalveolar Lavage
**BCC**	Basal cell carcinoma
**BCG**	Bacille Calmette-Guérin
**BCG SSI**	BCG Danish strain 1331 produced by Statens Serum Institute
**β-HCG**	Beta - Human Chorionic Gonadotrophin
**CCVTM**	Centre for Clinical Vaccinology and Tropical Medicine
**CFU**	Colony-forming unit
**CI**	Chief Investigator
**CIS**	Carcinoma *in situ*
**CRF**	Case Report Form
**DGE**	Differential Gene Expression
**DNA**	Deoxyribonucleic Acid
**ELISA**	Enzyme-linked immunosorbent assay
**ELISpot**	Enzyme-linked Immunospot
**FBC**	Full Blood Count
**GCP**	Good Clinical Practice
**GP**	General Practitioner (Family Doctor)
**HBsAg**	Hepatitis B Surface Antigen
**HBV**	Hepatitis B Virus
**HCV**	Hepatitis C Virus
**HIV**	Human Immunodeficiency Virus
**HLA**	Human Leukocyte Antigen
**ICH**	International Committee on Harmonisation
**IFN-ɣ**	Interferon Gamma
**IGRA**	Interferon-Gamma Release Assay
**IM**	Intramuscular
**LFT**	Liver Function Test
**LSC**	Local Safety Committee
**MAIT cell**	Mucosal associated invariant T cell
**MGIA**	Mycobacteria Growth Inhibition Assay
**MHRA**	Medicines & Healthcare Regulatory Authority
M.tb	*Mycobacterium tuberculosis*
**MVA**	Modified vaccinia Virus Ankara
**NK cells**	Natural Killer cells
**NHS**	National Health Service
**NIHR**	National Institute for Health Research
**OUH**	Oxford University Hospitals
**PBMC**	**Peripheral Blood Mononuclear Cells**
**PCR**	Polymerase Chain Reaction
**REC**	Research Ethics Committee
**SAE**	Serious Adverse Event
**SAR**	Serious Adverse Reaction
**SMC**	Safety Monitoring Committee
**SmPC**	Summary of Product Characteristics
**SOP**	Standard Operating Procedure
**SUSAR**	Suspected Unexpected Serious Adverse Reaction
**TB**	Tuberculosis
**TMF**	Trial Master File

5 BACKGROUND AND RATIONALE

5.1 Context

Mycobacterium tuberculosis (*M.tb*) is a pathogen with worldwide preponderance that infects humans and causes the transmissible disease tuberculosis (TB). An estimated one-third of the world's population is latently infected with *M.tb*, carrying a 10% lifetime risk of developing active life-threatening disease (1). In 2016, there were 10 million new cases worldwide and 1.7 million people died of TB (2). Co-infection with human immunodeficiency virus (HIV) greatly increases the risk of TB reactivation and death (3, 4). Diagnosis is challenging and drug treatment is often harmful, costly and complex. For these reasons, it is essential to develop a more effective vaccine against TB.

The Bacille Calmette-Guérin (BCG) vaccine is the only licensed *M.tb* vaccine and has been administered globally to several billion people over a 90 year period, by the intradermal route (5). Although it is effective in preventing disseminated TB disease including tuberculous meningitis in childhood, it does not protect against pulmonary TB in endemic areas (4, 6, 7).

Recent advances in TB vaccine development have primarily been in the area of viral-vectored vaccines, given in prime-boost regimes with BCG as the priming vaccine. The most advanced of these vaccine candidates, MVA85A, had promising phase I results in the UK when given by the intradermal route, but subsequently showed significantly lower immunogenicity in phase II efficacy trials in South Africa (8, 9). The failure of the MVA85A vaccine to improve efficacy in BCG-vaccinated infants and adults highlights our inability to predict which candidate TB vaccines might work in humans. The predictive value of preclinical animal models remains uncertain, and we do not have a validated immunological correlate of protection with which to guide vaccine design and the selection of which candidate vaccines should progress to efficacy trials.

An improved understanding of the nature of protective immunity in humans would significantly improve rational vaccine development. Whilst host immunity, particularly systemic adaptive immunity, has been well characterised in murine models, our understanding of the immunological events that occur in humans during acute infection is limited. In particular, our knowledge of human mucosal responses to *M.tb.* is limited. This is primarily due to the difficulties in studying early disease processes in the lung. Consequently, the majority of human studies have investigated immune responses *ex-vivo* in peripheral blood or after *in-vitro* infection of cell lines (10). A better understanding of the adaptive immune components that exist at the respiratory mucosal surfaces in humans could lead to interventions that prevent infection at the point of entry (11).

It is, however, well established that in both humans and mice, an effective Th1 cell-mediated immune response, characterised by the secretion of IFN-ɣ and TNF from antigen-specific CD4+ T-cells, is necessary but insufficient for protective immunity (12-17). Other components of host adaptive immunity for which there is some evidence of a role in protection include MHC class I-restricted CD8+ T-cells, CD1-restricted T-cells, IL-17 secreting T-cells and antibodies (18-21).

An innate immune response is essential for protective immunity (22). Up to 48% of people who are heavily exposed to *M.tb* do not develop any evidence of immune sensitisation (23). Innate immune cells such as dendritic cells, neutrophils and natural killer (NK) cells all play a role in the control of mycobacterial infection (24-26), as might tissue-resident T-cells, such as mucosal-associated invariant T-cells (MAIT), which possess immediate effector functions such as cytokine expression and cytotoxicity (27). Some of these immune pathways differ between animal models and humans. For example, humans express group 1 CD1a,b and c molecules, which present foreign lipid antigens (28). These molecules do not express in mice. Additionally, gamma delta T-cells are more important in the non-human primate model than in mice (29, 30).

The innate response also shapes the subsequent adaptive response. We found that TLR-1 expression on the day of vaccination predicted the magnitude of the cell-mediated immune response to a candidate TB vaccine (31). BCG vaccination resulted in NOD2-dependent epigenetic changes in monocytes which then enhanced cytokine production and increased non-specific pathogen clearance *in-vitro*, suggesting that innate immunity can be trained (32).

Although it is clear that the innate response is necessary for protective immunity, the balance, timing and coordination needed for an effective innate immune response in humans is unknown (11).

Challenge models

Observational field studies are of limited value in the study of human protective immunity, not least because the timing of exposure cannot be defined. Controlled human infection studies utilised in studies related to malaria, for example, to define the immunology and identify immune mechanisms of protection. Similar studies are now needed in TB to determine whether observations in the murine model translate to humans and to determine which vaccines and interventions will be effective in preventing human TB (33). These studies should focus on the mucosal route of infection (the lung) because systemic and lung immune responses may differ. It is not, however, ethical to administer virulent *M.tb* to humans, but BCG, a live replicating mycobacterial strain, is very closely related to *M.tb,* and is suitable for use in a human infection model. BCG elicits a similar host gene expression signature to *M.tb* (O'Shea unpublished data; Figure 1), and genetic studies demonstrate only 61 (of 3924) open reading frames (ORFs) present in *M.tb* are absent in BCG (34).

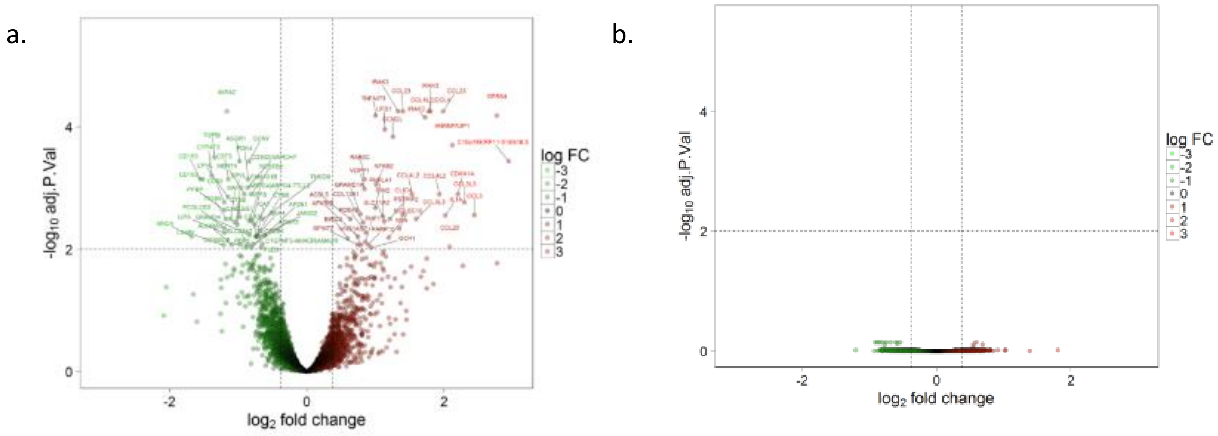

Figure 1. Changes in gene expression after PBMC from BCG-vaccinated subjects are stimulated with BCG or M.tb strain H37Rv. (a) BCG/H37Rv vs unstimulated; (b) BCG vs H37Rv.

Mycobacterial growth inhibition assay and correlates of protection

We have optimised a functional mycobacterial growth inhibition assay (MGIA) in which the degree of *M.tb* growth inhibition *in-vitro* in splenocytes from BCG-vaccinated and naïve mice directly correlates with the protective effect of BCG against *M.tb* infection *in-vivo* (35). BCG growth in PBMC from BCG-vaccinated non-human primates inversely correlates with lung pathology (Tanner, unpublished data; Figure 2). Furthermore, both whole blood and PBMC from recently BCG-vaccinated volunteers inhibit BCG growth *in-vitro* (36). This MGIA offers a controlled and tractable system that should allow us to identify the critical protective components of host immunity.

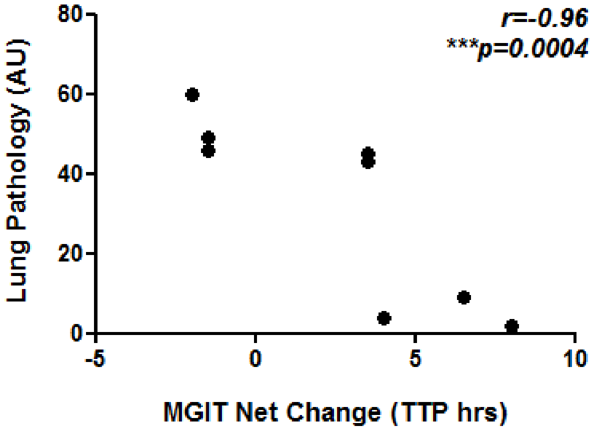

Figure 2. Net change in growth inhibition following BCG vaccination of non-human primates compared with lung pathology following in vivo challenge with M. Tb; (TTP: Time To Positivity; AU: Arbitrary units)

Basis for the aerosol inhaled route

A limitation of the BCG challenge work conducted to date is the intradermal route of delivery (37). The natural route of infection for *M.tb* is by inhalation of aerosolised infectious droplets containing tubercle bacilli, leading to the establishment of primary infection in the lung. The lung has a distinct mucosal immune system which is well adapted to encounter and process antigens. (38) Administering BCG challenge via the airway should therefore have the advantage over other routes of more correctly reflecting the mucosal response to infection with *M.tb*. This will aid our understanding of the evolution of early mycobacterial infection in the lung.

The inhaled route is a well-established route of drug delivery. Aerosolised droplets of broncho-dilating, anti-inflammatory and antimicrobial drugs are administered by inhalation to patients worldwide (39, 40). In the 1960s, BCG was safely delivered by aerosol in a small study in healthy subjects and in two studies in patients with lung cancer (41, 42).

We have established a new experimental medicine paradigm in which aerosolised BCG or candidate TB vaccines are delivered to the lungs. This is followed by bronchoscopic assessment of mucosal immunity. We have conducted 127 bronchoscopies on 91 healthy human subjects, of whom 22 have received aerosolised BCG in a dose-finding safety study without clinically significant adverse events ((43); clinicaltrials.gov NCT02709278, NCT01954563, NCT02532036). We are therefore well-placed to use state-of-the-art immuno-monitoring to dissect the human pulmonary innate and adaptive immune responses to aerosol BCG challenge.

Aerosol delivery devices

Currently the World Health Organisation (WHO) is making a major investment in developing new aerosol devices, providing a portable, low cost method of vaccine delivery (44). These devices are small, lightweight and aerosolise through a mesh to provide small and consistent particle size for vaccine or challenge agent delivery to the distal airway mucosa more accurately and precisely than conventional jet mechanisms.

One such mesh nebuliser is the MicroAIR NE-U22 (Omron® Healthcare Limited, Japan) which uses ultrasound to push liquid through a fine metal mesh. This generates an aerosol mist with a particle diameter of about 4µm. It is in current use with licensed drugs such as bronchodilating and antimicrobial agents (e.g. salbutamol, ipratropium bromide and tobramycin) and achieves good

bioavailability. BCG particles are typically around 2-4µm in size and can therefore be aerosolised with minimal damage (45-47). We have used this device in our previous human aerosol TB vaccine clinical trials, TB026 and TB035 (43); Clinicaltrials.gov NCT01954563), and the current TB041 clinical trial (Clinicaltrials.gov NCT02709278) with a good usability and safety profile.

TB041: A clinical challenge trial to evaluate controlled human infection with BCG administered by the aerosol-inhaled route compared with the intradermal route in healthy, BCG-naïve, UK adult volunteers. Clinicaltrials.gov NCT02709278

TB041 is an ongoing trial in our group that delivers BCG via the aerosol route. The aims of the study are to determine tolerability of BCG delivered by this route, and feasibility as a challenge model, by assessing BCG recovery from the BAL fluid. Volunteers undergo bronchoscopy with bronchoalveolar lavage 14 days following inhalation of BCG. The study initially used BCG SSI, the licenced BCG vaccine in the UK for intradermal vaccination, and 10 volunteers inhaled BCG SSI with no safety concerns. In 2016 Statens Serum Institute ceased production of BCG SSI Danish strain. A substantial amendment was submitted and approved by MHRA and OxREC A, to start a new arm of the study using BCG Bulgaria, the BCG vaccine recommended for use in the UK by Public Health England (48).

We have now delivered BCG Bulgaria via the aerosol route to 12 volunteers and repeated the dose escalation with no safety concerns (Appendix A). Three volunteers received $1x10^4$cfu BCG Bulgaria; three volunteers received 1 $x10^5$cfu, three volunteers received 1×10^6cfu, and three volunteers have received the target dose of 1×10^7cfu BCG Bulgaria via aerosol delivery.

We are currently completing enrolment into the blinded paired placebo groups, whereby volunteers randomised to aerosol-inhaled BCG receive a concurrent intradermal saline injection, while volunteers given intradermal BCG receive a concurrent dose of inhaled saline. This design allows a distinction to be made between any adverse events attributable to the method (including nebuliser device) of challenge delivery and those attributable to the inhaled BCG challenge itself.

Bronchoscopy and bronchoalveolar sampling

Obtaining BAL samples allows us to evaluate the local innate and adaptive immune response induced by aerosol BCG. This also allows us to determine whether we can detect BCG using culture and PCR from the BAL fluid. Protection from TB induced by intra-pulmonary vaccination is associated with cellular immune responses detected in the BAL samples of immunised mice (49). In macaques receiving aerosolised MVA85A, we detected significant levels of antigen-specific cellular immune responses in BAL samples (50), and in 12 UK adults who received aerosol MVA85A, we also detected significant levels of antigen-specific cellular immune responses in these BAL specimens, with higher frequency compared to adults who received intradermal MVA85A (43). Both CD4+ and CD8+ antigen-specific T-cells were detectable in the BAL. In macaques receiving aerosolised BCG, poly-functional T-cells and significant levels of antigen-specific cellular immune responses were detected in BAL specimens (47). These studies indicate the utility of BAL samples in determining correlates of protection.

BAL samples are obtained by performing a bronchoscopy, a widely and safely used procedure. The short procedure involves the insertion of a narrow flexible fibre-optic tube into the airway under light intravenous sedation and topical local anaesthesia. Under direct vision, saline is delivered to a section of lung mucosa and then recollected by suction. In all clinical trials using bronchoscopy, TB026 (43), TB035 and TB041 (Clinicaltrials.gov NCT01954563 and NCT02709278) bronchoscopies have been well tolerated by volunteers. During bronchoscopy other samples may be taken such as using a swab to absorb airway fluid ("bronchosorption") or bronchial brushings. Collecting these samples will not impact on total length of the bronchoscopy procedure, the risk to the volunteer or the overall experience by the volunteer of the bronchoscopy.

Cellular immune responses from BAL only describe immune responses in the mucosa and airways and not the lung parenchyma. It is now well recognised that localisation of immune cells into distinct compartments through homing markers influences protective immunity. In patients with active TB, peripheral blood is depleted of *M.tb*-specific MAIT cells while BAL is enriched (51). In murine adoptive transfer studies, $CXCR3^{hi}CD4+$ T-cells preferentially localise to the lung parenchyma and are better at controlling *M.tb* infection than their $CX3CR1^{hi}KLRG1^{hi}$ counterparts which localise to the lung intravasculature (52). Therefore, in this study, we will also obtain lung tissue samples by endobronchial biopsy during the bronchoscopy. This is a routine procedure where forceps on the end of a wire are passed into the airway and a small sample is collected. Due to the small size of the biopsy this procedure results in minimal bleeding, and we foresee it to be no less tolerated than performing a bronchoscopy with BAL only as in previous studies (53, 54). Importantly this procedure will allow us to characterise for the first time the motility and extravasation of immune cells to the local site of infection.

Data from human BCG aerosol studies are lacking; therefore, time points have been extrapolated from human TB viral vector aerosol studies (MVA85A) or animal BCG aerosol studies to capture the immune response as broadly as possible.

5.2 Rationale

Hypothesis

We hypothesise that following Bacille Calmette-*Guérin* (BCG) aerosol challenge, components of the innate immune response will correlate with the adaptive immune response and subsequent protection as demonstrated by *in-vitro* PBMC mycobacterial growth inhibition at Day 56.

BCG and dosage

We will deliver a dose of $1x10^7$cfu BCG Bulgaria by the aerosol route. This is the same dose that was used for Arm 2 of TB041. TB041 is still open for enrolment. Preliminary safety data is shown in Appendix A.

Rationale for dose

The intradermal licensed dose of BCG Bulgaria for adults is 1.5-6.0 x 10^5cfu in 0.1ml (55).

We calculated the dose for TB043 based upon an average of this: $4x10^5$cfu in a 0.1ml adult standard dose, which translates into a BCG concentration of $4x10^6$cfu/ml.

We aim to deliver this standard dose of $4x10^5$cfu into the lung by loading $1x10^7$cfu BCG into the nebuliser. This accounts for the losses of BCG due to method of delivery as follows:

1. The amount of viable BCG cfu recoverable from a vial of BCG is often lower than the expected amount based on the Summary of Product Characteristics (SmPC). The viable BCG cfu from the vials of BCG Bulgaria reconstituted in the trial to date is shown below (Figure 3). Many vials are one-half to one log lower than the 1.5-$6x10^6$cfu/ml stated in the SmPC.

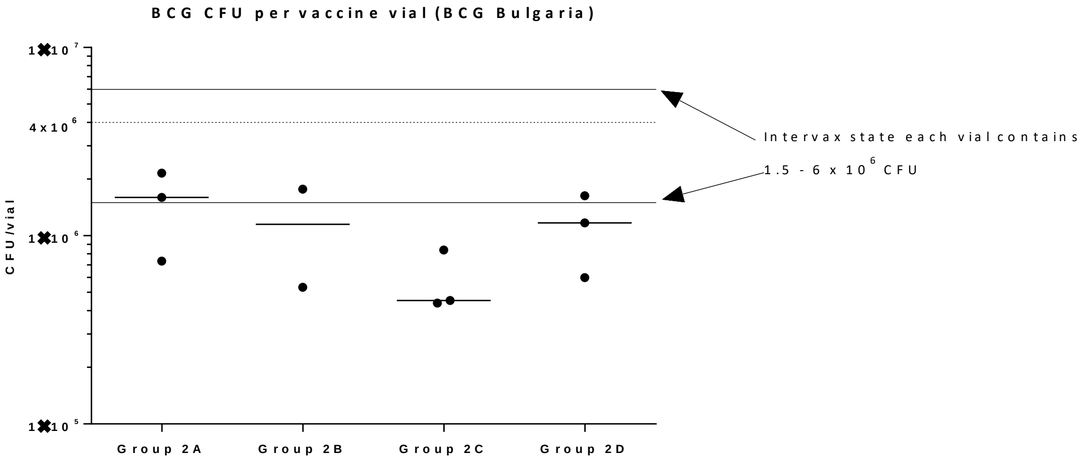

Figure 3. Viable BCG cfu from BCG Bulgaria vials reconstituted in the TB041 trial to date

2. The vaccine vial is diluted to achieve the required dose and we have plated out these dilutions to obtain an estimate of the dose that is loaded onto the nebuliser (*Figure* ***4***). This loaded dose is consistently one-half to one log lower than our intended dose.

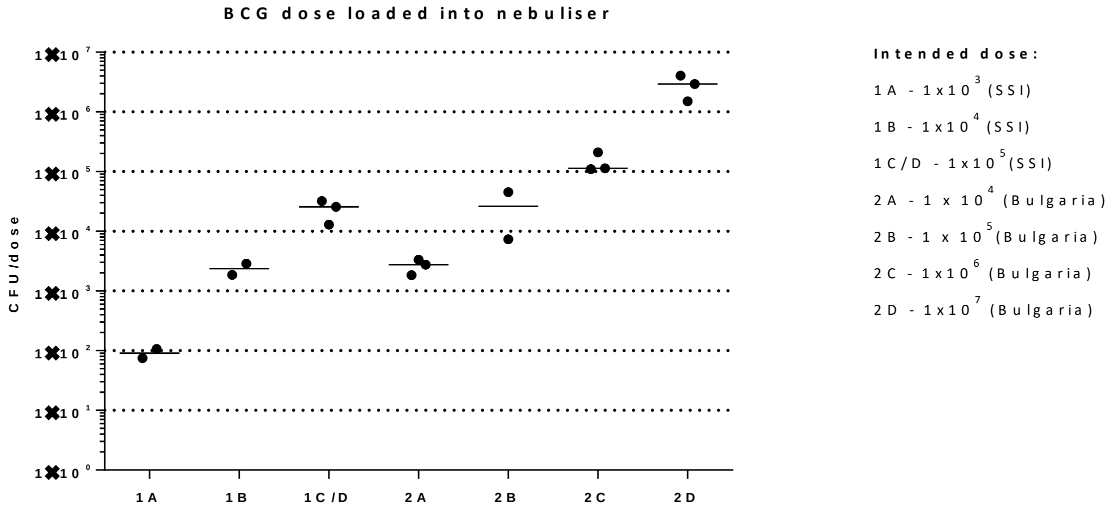

Figure 4. BCG cfu intended dose following dilution of either BCG SSI and BCG Bulgaria

3. We have developed an *in-vitro* model to quantify the actual delivered dose of nebulised BCG. This involved using a vacuum pump attached to an impinger to collect the aerosol expelled by the Omron nebuliser. The amount of BCG in this collected sample was quantified by culture plating. This model consistently measured an approximately 50% loss of BCG compared to the amount of BCG loaded into the nebulizer (*Figure* ***5***). This model does not take into account the further potential loss of BCG that occurs in a clinical setting through loss of aerosol into the environment, as the volunteer does not breathe in continuously, and the nebuliser continues to aerosolise the BCG between breaths.

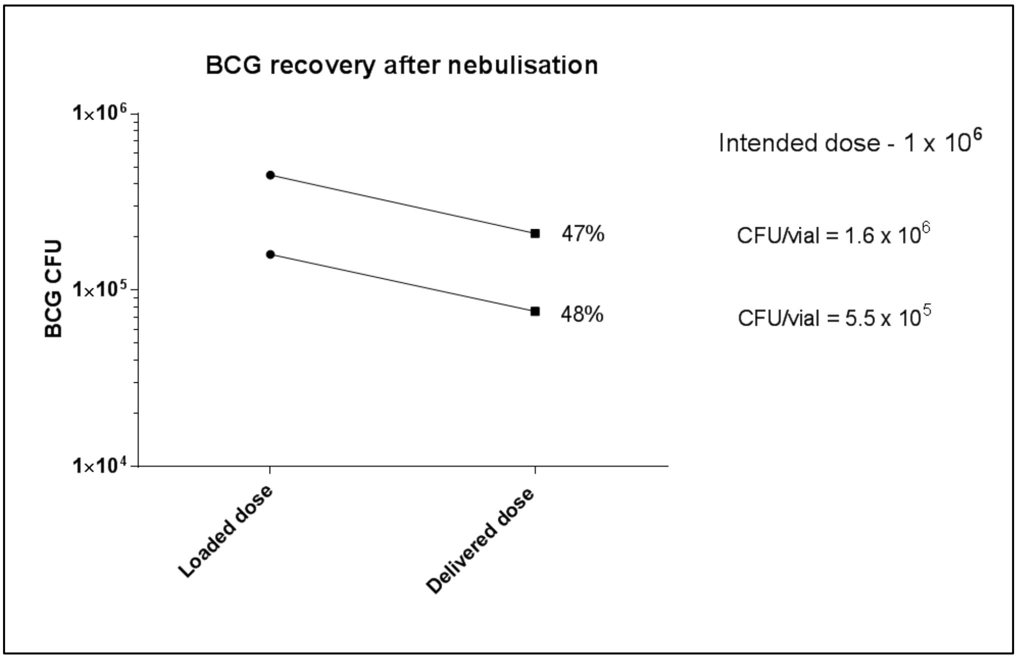

Figure 5. BCG recovery after nebulisation using vacuum model

Given these documented losses, we ensure the appropriately delivered dose of ~$4x10^5$cfu by utilising a prepared dose of $1x10^7$cfu.

5.3 Risks and Benefits

Potential risks

The potential risks to participants in this study include risks associated with:

1. Venepuncture and intravenous cannulation

Localised bruising and discomfort can occur at the site of venepuncture. Infrequently fainting may occur. The total volume of blood drawn over a six-month period will be 520ml which should not compromise these otherwise healthy volunteers, as they would donate 470mL during a single blood donation for the National Blood transfusion Service over a 3-4 month period. Volunteers will be asked to refrain from blood donation for the duration of their involvement in the study.

An intravenous cannula is routinely inserted into a peripheral (usually forearm) vein prior to bronchoscopy in order to administer intravenous sedation. This is removed once the procedure is completed. The risks of cannulation are identical to those associated with venepuncture but include an additional small risk of soft tissue infection. This risk will be minimised by an aseptic insertion technique and is easily recognisable and treatable. The short duration of cannulation (a few hours) further minimises this risk.

2. BCG challenge

BCG Bulgaria is on the WHO list of pre-qualified vaccines and has a well-defined side effect profile. Full details are given in the SmPC (55). BCG is licensed for delivery via the intradermal route. It is not licensed for delivery via the aerosol route.

Anticipated local adverse events following aerosol challenge include mild throat discomfort and cough. Transient bronchospasm causing wheezing, shortness of breath, chest tightness or chest pain are also possible (Appendix A).

Systemic reactions

Systemic reactions to aerosolised BCG could include transient fever or feverishness, malaise, nausea, headache, myalgia, arthralgia or fatigue. In the aerosol BCG trial TB041 (Clinicaltrials.gov NCT02709278), approximately 1/3 of participants experienced systemic symptoms, with an average duration of 24 hours.

Following intradermal BCG, disseminated complications of BCG, such as bone infections, have been reported, but are extremely rare and usually reported in immunocompromised individuals. No such complications have occurred in any trial delivering aerosolised BCG. Volunteers are screened to ensure they are immunocompetent prior to enrolment. BCG Bulgaria is fully susceptible to isoniazid, rifampicin, ethambutol and all second line anti-tuberculous drugs (56).

Allergic reactions

Allergic reactions from mild to severe may occur in response to any constituent of a medicinal product's preparation. Anaphylaxis is extremely rare (less than 1/1000 people) but can occur in response to any vaccine or medication.

3. Bronchoscopy

Bronchoscopy is a widely and safely used investigative procedure in clinical research studies involving both healthy volunteers and patients with respiratory conditions such as asthma and interstitial lung disease (57, 58). Clinical guidelines for performing investigative bronchoscopy in research studies are well established (59).

The bronchoscopies will be carried out in a dedicated NHS bronchoscopy suite with an excellent safety record by highly skilled and experienced consultant respiratory physicians. Intravenous sedation and topical local anaesthesia are administered prior to bronchoscopy to reduce discomfort, facilitate the procedure, and remove memory of the event. (In 98% of cases, subjects have no memory of the procedure). Trained, experienced staff and facilities for resuscitation and drugs for reversal of sedation will be available. To further minimise risk, volunteers will be excluded if they have an abnormal chest radiograph, increased bleeding risk, a significant smoking history, a clinically significant history of atopy or any evidence of lung disease, including asthma (as defined by: a clinical diagnosis of asthma; prescription of asthma medication; airflow obstruction on spirometry; history of nocturnal or exercise-induced wheeze).

The risks of bronchoscopy are discussed at the time of consent and comprise: adverse reaction to sedation or local anaesthetic, sore throat and /or transient hoarse voice, blood-stained phlegm, laryngospasm/bronchospasm, hypoxia, post-procedure flu-like symptoms (1-2 days), damage to airways and risk of death (<1 in 100,000).

- Bronchoalveolar lavage and endobronchial biopsy are routine procedures in investigative bronchoscopy and carry a minimum bleeding risk. The risk of infection or febrile reactions will be minimised by full bronchoscope asepsis.
- Respiratory depression secondary to sedation is rare. No rescue medication for oversedation has been required for volunteers participating in any of our aerosol clinical trials to date (43)), Clinicaltrials.gov NCT02532036, NCT01954563 and NCT02709278).
- Allergic reactions from mild to severe may occur in response to any constituent of the local anaesthetic or sedative agents. Anaphylaxis is extremely rare but can occur. The Summary of Product Characteristics (SmPC) for the local anaesthetic and sedative agents contain full details of the indications and side effects of these licensed medications.

4. Chest radiograph

A chest radiograph is a painless radiological investigation that exposes volunteers to approximately 0.02 milliSieverts of radiation, equivalent to around 3 days of natural background radiation. The additional risk of cancer due to one chest radiograph is insignificant (1/900,000).

5. Spirometry

Vigorous respiratory manoeuvres such as forced expiration through the spirometer can occasionally lead to coughing or light-headedness, but these symptoms are mild and rapidly self-limiting.

6. Induced sputum

Inhalation of hypertonic saline to induce sputum production can occasionally result in excessive bouts of coughing or sensation of shortness of breath but these symptoms are mild and rapidly self-limiting. Rarely, hypertonic saline can induce bronchoconstriction but this is very unlikely to occur in our cohort of patients who have no clinically significant history of atopy and no asthma. Hypertonic saline-induced bronchospasm is quickly reversed by treatment with an inhaled short-acting B_2 agonist. Volunteers will be pre-treated with nebulised salbutamol and their lung function will be monitored throughout the procedure. This procedure has been used in TB041 Clinicaltrials.gov NCT02709278 with no adverse events and has been well tolerated by volunteers.

Potential benefits

Volunteers are not expected to benefit directly from participation in this study. Volunteers will gain some information about their general health as a result of the screening history, examination, blood tests, urine tests, chest radiograph and spirometry. They may also gain health information from the bronchoscopy. Volunteers found to have a previously undiagnosed condition thought to require further medical attention will be referred appropriately to their GP or an NHS specialist service for further investigation and treatment, with their permission.

It is hoped that their contribution will further the development of a safe and successful vaccine for TB and our knowledge about TB infection and protection.

6 OBJECTIVES AND OUTCOME MEASURES

Table 2. Objectives and Outcome Measures

Objectives	Outcome Measures	Timepoint(s) of evaluation of this outcome measure
**Primary Objective**<br>To define the systemic and mucosal innate and adaptive immune responses induced by aerosol inhaled BCG infection in healthy BCG and *M.tb* naïve UK adults	Laboratory markers of innate and adaptive immunity, including *ex-vivo* ELISpot and ELISAs; and RNA sequence analysis and intracellular cytokine staining or chip cytometry with blood, BAL and biopsy samples	At each visit
**Secondary Objective**<br>To identify laboratory markers of the immune response that correlate with protection as defined by PBMC MGIA at D56 post challenge	Established and exploratory markers of innate, cell mediated and humoral immunity in blood, BAL and biopsy samples; Culture (colony counting) and PCR quantification of BCG in BAL; MGIA on PBMCs collected at Day 56	At each visit

**Tertiary Objective**<br>To describe the human clinical response to BCG challenge by the aerosol inhaled route in healthy, BCG-naïve UK adult volunteers	Actively and passively collected data on adverse events; detailed participant symptom profiles; Laboratory parameters including BCG CFU counts in induced sputum	At each visit and via e-diary(s)

7 PARTICIPANT IDENTIFICATION

7.1 Study Participants

Healthy, BCG and *M.tb.*-naïve UK adults have been chosen because of their low baseline level of anti-mycobacterial immunity. The aim is to identify a primary innate and adaptive immune response after aerosol BCG infection in people who are mycobacterially naïve.

Inclusion Criteria

Volunteers must meet all of the following criteria to enter the study:

- Healthy adult aged 18-50 years
- Resident in or near Oxford for the duration of the study period
- Screening IGRA negative
- No relevant findings in medical history or on physical examination
- Allow the Investigators to discuss the individual's medical history with their GP
- Use effective contraception (see below) for the duration of the study period (females only)
- Refrain from blood donation during the study
- Give written informed consent
- Allow the Investigator to register volunteer details with a confidential database (The Over-volunteering Protection Service) to prevent concurrent entry into clinical studies/trials
- Able and willing (in the Investigator's opinion) to comply with all the study requirements

Exclusion Criteria

Volunteers must meet none of the following criteria to enter the study:

- Previously resident for more than 12 months concurrently in a tropical climate where significant non-tuberculous mycobacterial exposure is likely
- Participation in another research study involving receipt of an investigational product in the 30 days preceding enrolment, or planned use during the study period[a]
- Prior vaccination with BCG or any candidate TB vaccine
- Administration of immunoglobulins and/or any blood products within the three months preceding the planned study challenge date
- Clinically significant history of skin disorder, allergy, atopy, immunodeficiency (including HIV), cancer (except BCC or CIS), cardiovascular disease, gastrointestinal disease, liver disease, renal disease, endocrine disorder, neurological illness, psychiatric disorder, drug or alcohol abuse
- Concurrent oral, inhaled or systemic steroid medication or the concurrent use of other immunosuppressive agents

- Shares a household with someone with clinically significant immunodeficiency (either from infection or medication) who is deemed to be at risk of developing disseminated BCG infection if exposed to BCG
- History of anaphylaxis to vaccination or any allergy likely to be exacerbated by any component of the study agent, sedative drugs, or any local or general anaesthetic agents
- Pregnancy, lactation or intention to become pregnant during study period
- Any respiratory disease, including asthma
- Current smoker (defined as any smoking including e-cigarettes in the last 3 months)
- Clinically significant abnormality on screening chest radiograph[b]
- Clinically significant abnormality of spirometry
- Any nasal, pharyngeal, or laryngeal finding which precludes bronchoscopy
- Current use of any medication taken through the nasal or inhaled route including cocaine or other recreational drugs
- Clinical, radiological, or laboratory evidence of current active TB disease
- Past treatment for TB disease
- Any clinically significant abnormality of screening blood or urine tests[b]
- Positive HBsAg, HCV or HIV antibodies[b]
- Any other significant disease, disorder, or finding, which, in the opinion of the Investigator, may either put the volunteer at risk, affect the volunteer's ability to participate in the study or impair interpretation of the study data

[a] Volunteers will be excluded from the study if they are concurrently involved in a study or trial that involves regular blood tests or an investigational medicinal product. In order to check this, volunteers will be asked to provide their National Insurance or Passport number (if they are not entitled to a NI number) and will be registered on a national database of participants in clinical trials (www.tops.org.uk).

[b] Volunteers who are excluded from the study because they have been discovered during screening procedures to be suffering from a previously undiagnosed condition thought to require further medical attention will be referred appropriately to their GP or an NHS specialist service for further investigation and treatment.

Effective contraception for female volunteers

Female volunteers are required to use an effective form of contraception during the course of the study.

Acceptable forms of contraception for female volunteers include:

- Established use of oral, injected on implanted hormonal methods of contraception
- Placement of an intrauterine device (IUD) or intrauterine system (IUS)
- Permanent sterilisation or bilateral tubal occlusion
- Barrier methods of contraception (condom; or occlusive cap with spermicide)
- Male sterilisation, if the vasectomised partner is the sole partner for the subject
- True abstinence, when this is in line with the preferred and usual lifestyle of the subject (periodic abstinence and withdrawal are not acceptable methods of contraception)

8 STUDY DESIGN AND PROCEDURES

This is a randomised controlled clinical challenge study of BCG administered by the aerosol-inhaled route in healthy UK volunteers.

8.1 Study Numbers and Groups

65 volunteers will be enrolled; the first thirteen to Group 1, the next thirteen to Group 2, the next thirteen to Group 3, the next thirteen to Group 4 and the final thirteen to Group 5. Once allocated to groups, the volunteer will be randomly allocated to either BCG challenge (Arm A) or placebo saline control (Arm B); Table 3.

In the event of bronchoscopy postponement, (see postponement criteria 8.10) the bronchoscopy will be rescheduled where possible within the allowable window for that follow up visit. If possible the rest of the procedures for that follow up visit will occur on the scheduled day (e.g. observations, e-diary check, blood sample collection), with the bronchoscopy to follow. If the bronchoscopy occurs outside of the allowable time window (as is likely for deferred bronchoscopies in Groups G1, G2 or G3 due to the tight time windows) the volunteer may be reallocated to a later Group in order to fit in with schedule, as per the discretion of the investigator. A new volunteer will then be enrolled into that original group to replace the volunteer whose bronchoscopy was postponed.

Table 3. Study groups

Group	Randomised Intervention	Sample size
**Group 1:** Bronchoscopy Day 2 post challenge	**Arm A BCG**: Aerosol inhaled BCG Bulgaria	10
	Arm B Control: Aerosol inhaled normal saline placebo	3
**Group 2:** Bronchoscopy Day 7 post challenge	**Arm A BCG**: Aerosol inhaled BCG Bulgaria	10
	***Arm B Control*:** Aerosol inhaled normal saline placebo	3
**Group 3**: Bronchoscopy Day 14 post challenge	**Arm A BCG**: Aerosol inhaled BCG Bulgaria	10
	Arm B Control: Aerosol inhaled normal saline placebo	3
**Group 4:** Bronchoscopy Day 28 post challenge	**Arm A BCG**: Aerosol inhaled BCG Bulgaria	10
	Arm B Control: Aerosol inhaled normal saline placebo	3
**Group 5**: Bronchoscopy Day 56 post challenge	**Arm A BCG**: Aerosol inhaled BCG Bulgaria	10
	Arm B Control: Aerosol inhaled normal saline placebo	3

8.2 Recruitment

Volunteers may be recruited by use of an advertisement formally approved by the ethics committee and distributed or posted in the following places:

- In public places (including NHS hospitals and university buildings) with the agreement of the owner or proprietor
- In newspapers or other literature for circulation
- On radio via announcements
- On a website operated by our group or with the agreement of the owner or operator
- By e-mail distribution to a group or list only with the express agreement of the network administrator or with equivalent authorisation
- On stalls or stands at exhibitions or fairs

- Direct mail-out: This will involve obtaining names and addresses of adults via the most recent Electoral Roll. The contact details of individuals who have indicated that they do not wish to receive postal mail-shots would be removed prior to the Investigators being given this information. The company providing this service is registered under the Data Protection Act 1998. Investigators would not be given dates of birth or ages of individuals, but the list supplied would only contain names of those aged between 18-50 years (as per the inclusion criteria).
- Oxford Vaccine Centre databases: We may contact individuals from databases of groups within the CCVTM (including the Oxford Vaccine Centre database) of previous trial participants who have expressed an interest in receiving information about future studies for which they may be eligible.

A copy of intended advertising will be submitted with the initial application for ethical approval and any significant changes to this advertisement will be submitted as an amendment to the ethics committee for approval before use.

Volunteers who express an interest in the study will be given a Volunteer Summary Sheet and asked to register for the study through the website. In addition, and with their permission, their details will be passed to the study team to arrange a pre-screening call and screening appointment.

8.3 Informed Consent

Written informed consent will be obtained at screening by a GCP trained Investigator.

The volunteer must sign and date the latest approved version of the consent form before any study specific procedures are performed.

The Volunteer Information Sheet (VIS) will be made available to the volunteer no less than 24 hours prior to attending for screening. The details enclosed will be discussed with the volunteer, including:

- the aims and nature of the study
- the agents used
- the schedule of visits and tests to be carried out
- the implications and constraints of the protocol
- compensation for the volunteer
- known side effects and risks of taking part in the study

The following general principles will be emphasised:

- Participation is entirely voluntary
- The volunteer may withdraw from the study at any time for any reason
- Withdrawal or refusal to participate involves no penalty or loss of medical benefits
- The volunteer is free to ask questions at any time to allow him or her to understand the purpose of the study and the procedures involved
- There is no direct benefit from participating
- The volunteer's GP will be contacted to corroborate their medical history and confirm that the volunteer does not meet any of the exclusion criteria
- The volunteer will be registered on the TOPS database (The Over-Volunteering Protection System)
- With permission, the volunteer's samples taken as part of the study will be stored indefinitely for further research.

The volunteer will then have time to consider whether or not to participate. If the volunteer decides to participate, they will sign and date two copies of the consent form, one for them to take away and keep, and one for the Investigator that will be retained at the study site. These forms will also be

signed and dated by the Investigator. This will occur before any study specific procedures are performed.

8.4 Screening and Eligibility Assessment

Once the volunteer has given their consent to undergo screening, a baseline medical history (including concomitant medication and allergy to medication) and physical examination will be performed by a GCP trained doctor. Inclusion and exclusion criteria (listed below) will be checked using a tabulated format. Demographic and occupational data will be collected. Vital signs will be checked and bloods taken including FBC, clotting profile, U&Es, LFTs, HIV antibodies, HBsAg, HCV antibodies and IGRA. Spirometry will be performed according to local SOP. Volunteers will be counselled by one of the Investigators for HIV, Hepatitis B and Hepatitis C testing. Urine will be tested for the presence of clinically significant proteinuria, glycosuria or haematuria. A pregnancy test will be performed for female volunteers. Volunteers will then attend for a chest radiograph.

Laboratory parameters for inclusion/exclusion in the study will be considered on an individual basis, with Investigator discretion for interpretation of results and the need for repeated tests. In general, volunteers will be excluded if a result at screening constitutes what would qualify as a grade 1 (or higher) laboratory AE, according to the laboratory AE tables found in the relevant SOP and filed in the trial master file (TMF).

The total duration of the screening visit will be around 2 hours. Depending upon availability and scheduling of the volunteer and NHS trust radiography staff, the screening may be split across two visits.

8.5 Randomisation and blinding

Volunteers will be allocated to a study group (Groups 1 - 5) in a sequential manner. To reduce bias and the influence of any difference in the baseline characteristics between the BCG and control groups, we will randomise the allocation of volunteers using sequentially numbered sealed envelopes, prepared by an independent statistician at the Department of Primary Care.

Volunteers will be blinded to eliminate subject bias (either conscious or subconscious) using a 10:3 randomised control design (BCG:placebo) whereby volunteers randomised to the BCG arms (Arm A) will inhale aerosolised BCG mixed with normal saline and those randomised to the placebo arms (Arm B) will inhale aerosolised normal saline. This design has the added benefit of allowing a distinction to be made between any adverse events attributable to the method (including nebuliser device) of challenge delivery and those attributable to the inhaled challenge product itself. The volunteers will be unblinded just prior to the 3 month visit when those in Arm A will have sputum collected and those in Arm B will not.

The bronchoscopist performing the procedure will also be blinded to eliminate any bias in the reporting of the appearance of the lung mucosa and extent of airway inflammation.

All samples will be anonymised and the subject number will be allocated sequentially and therefore not identifiable with the allocated Arm. The senior immunologist will be blinded to reduce any bias that could be introduced at the sample processing stage.

8.6 Follow up visits

D0 - Challenge

Any new medical issues or symptoms that have arisen will be assessed. Venepuncture and other samples will be taken according to the Schedule of Study Procedures in section 3.2. Vital signs and spirometry will be performed. The inclusion and exclusion criteria for the study will be reviewed;

provided that the volunteer still satisfies all inclusion criteria (and no exclusion criteria) and their consent remains valid, the volunteer will be allocated to a group and randomised to study intervention. BCG challenge or placebo will be administered by aerosol using the Omron nebuliser according to the study-specific SOP. Volunteers will be kept under observation for around 60 minutes after challenge. The challenge visit will last approximately 3 hours. The e-diary will be set up during this visit.

E-diaries

All volunteers will complete an e-diary from Day 0 through to Day 13 following challenge. The e-diary will ask for recorded temperature and lung function (using a take-home spirometer) and will solicit respiratory and systemic symptoms as well as capturing any unsolicited symptoms. A second 7 day e-diary will be completed by volunteers in Groups 3-5, starting at time of bronchoscopy, in order to capture bronchoscopy-related symptoms. For Groups 1 and 2 this data will be captured within their first e-diary as their bronchoscopy will fall within the first e-diary period.

Bronchoscopy

The bronchoscopy visit lasts several hours and takes place at the bronchoscopy suite in the OUH NHS Foundation Trust by the specialist team there. Blood will be taken as specified in the Schedule of Study Procedures (Table 1). Details of the procedure are outlined in Section 5.3.

Other follow-up visits

These will be performed as specified in the Schedule of Study Procedures (Table 1). Any new medical issues or symptoms that have arisen will be assessed, and the e-diary (if applicable) and any ongoing adverse events will be reviewed. Additional procedures or laboratory tests may be carried out at the discretion of the Investigators if deemed clinically necessary. Follow-up visits will last approximately 15-30 minutes, except for D84 and D168 visits when induced sputum is collected for volunteers in Arm A. For these volunteers, the visit will last approximately 1.5 hours. The study duration will be approximately 24 weeks from the date of enrolment.

Volunteers will be advised of the intervention they received (BCG infection or saline control) prior to their 3 month visit (or earlier if they withdraw).

After the final study visit, an End of Study letter will be sent to each volunteer's GP to inform them that the volunteer has either completed the study and which intervention they received, or was not enrolled.

8.7 Timepoints

Screening – study enrolment time window

Enrolment should take place no longer than 120 days following the date of screening appointment. If more than 120 days elapse, the screening visit should be repeated in full, prior to enrolment, in order to minimise the risk to participants of any new unidentified health problems having arisen during that period.

Follow up period

The follow up period will be six months in accordance with findings from previous studies in which adequate safety data and reliable markers of immunogenicity have been obtained in this time interval.

8.8 Sample handling

Details regarding samples, volume and frequency of sampling are listed in the Schedule of Study Procedures (Table 1). Blood and other samples will be processed according to local laboratory SOPs. All samples will be anonymised at the CCVTM/OUH.

Volunteers will be informed that there may be leftover samples of their blood, BAL, biopsy and sputum. With the volunteers' informed consent, any leftover samples will be frozen for future analysis. This may include human DNA and RNA analysis to search for correlates of TB risk and/or protection. Samples may be shipped to other parties involved in our research in anonymised form for immunological analysis. Volunteers will be able to decide if they will permit such future use of any leftover samples. If they elect not to permit this, leftover samples will be discarded.

8.9 Challenge Postponement Criteria

Challenge will not proceed on the scheduled day in any of the following situations:

- The volunteer has a current or recent upper respiratory tract infection, unless they have been symptom-free for at least one week
- The volunteer has a temperature > 37.5^{o}C
- The Investigator judges the volunteer to have an acute moderate or severe illness (whether febrile or not)
- The volunteer has received a live vaccine within the preceding 28 days
- The Investigator has any other concern that challenge may not be in the volunteer's best interests

In these cases, the volunteer may be challenged at a later date or withdrawn from the study at the discretion of the Investigator.

8.10 Bronchoscopy Postponement criteria

Bronchoscopy will not proceed on the scheduled day in any of the following situations:

- The volunteer has a temperature > 37.5^{o}C
- The Investigator judges the volunteer to have an acute moderate or severe illness (whether febrile or not)
- The Investigator has any other concern that bronchoscopy may not be in the volunteer's best interests

8.11 Discontinuation / Withdrawal Criteria

Every reasonable effort will be made to maintain protocol compliance and participation in the study.

In accordance with the principles of the current revision of the Declaration of Helsinki and any other applicable regulations, a volunteer has the right to withdraw from the study at any time and for any reason, and is not obliged to give his or her reasons for doing so. The Investigator may withdraw the volunteer at any time in the interests of the volunteer's health and well-being (including on the advice of the LSC). In addition, the volunteer may withdraw/be withdrawn for any of the following reasons:

- Administrative decision by the Investigator
- Ineligibility (either arising during the study or retrospectively, having been overlooked at screening)
- Significant protocol deviation
- Volunteer non-compliance with study requirements (failure to attend two or more follow-up visits)
- Any AE which requires discontinuation of study involvement or results in inability to comply with study procedures

- Confirmed pregnancy during the study

Any volunteer who becomes pregnant during the study will be followed up as per the protocol and until the end of the pregnancy. We will not routinely perform venepuncture on a pregnant volunteer.

The reason for withdrawal will be recorded in the Case Report Form (CRF). Volunteers withdrawn from the study may be replaced on the decision of the Investigator. If the volunteer is withdrawn due to an AE, the Investigator will arrange for appropriate specialist management or follow up visits or telephone calls until the AE has resolved or stabilised. The extent of follow up after premature discontinuation will be determined by the Investigator but will be at least for the whole study period, and if pregnant, until pregnancy outcome. An 'End of Study letter' will be sent to each withdrawn volunteer's GP.

If a volunteer withdraws from the trial, samples collected before their withdrawal from the study will be used/stored unless the volunteer specifically requests otherwise. Long term safety data collection will continue as appropriate if a volunteer has received a challenge dose.

8.12 Safety

Discontinuation of the study

The study will be discontinued in the event of any of the following:

- New scientific information is published to indicate that volunteers in the study are being exposed to undue risks as a result of administration of BCG, or as a result of the study procedures or follow-up schedule.
- Serious concerns about the safety of BCG arise as a result of one or more challenge-related SAE(s) occurring in the volunteers enrolled in this or any other ongoing study of BCG.
- For any other reason at the discretion of the Investigator.

8.13 End of Study Definition

The study will be completed when the last volunteer enrolled into the study has completed their final follow up visit.

9 STUDY AGENTS AND DEVICES

9.1 BCG Description

BCG Bulgaria

BCG Bulgaria is a dried preparation containing live bacteria derived from an attenuated strain of *Mycobacterium bovis* BCG. It is supplied as a powder and diluent for suspension. Each vial contains 1.5-6x10^6cfu (see SmPC).

Storage of BCG

BCG Bulgaria will be shipped to the CCVTM, University of Oxford, Churchill Hospital, in the presence of a temperature logger. The vaccine will be re-labelled for local use.

Dispensed BCG vials are supplied in boxes containing multiple vials and each box is clearly labelled with the market product.

BCG will be stored at +2 to +8°C (nominal temperature) in a secure, temperature-monitored refrigerator at the CCVTM, University of Oxford, Churchill Hospital.

9.2 Dispensing and administration

All movements of vials of the study agent in or out of the locked refrigerator will be documented. BCG accountability, storage, shipment and handling will be in accordance with local SOPs and other relevant local forms.

BCG will be administered by aerosol inhalation, according to the study-specific SOP. Volunteers will stay in the unit for 60 minutes (±10 minutes) after challenge. During the administration of BCG, monitoring equipment, oxygen, medicines including bronchodilators and resuscitation equipment will be immediately available for the management of anaphylaxis and bronchospasm according to the study-specific SOP.

In order to minimise dissemination of the BCG bacteria into the environment and to ensure the protection of staff, measures will be instituted during and following challenge to fully comply with local infection control and Occupational Health regulations.

9.3 Saline

Sterile saline will be mixed with the reconstituted BCG to add to the nebuliser. Sterile saline will also be used for placebo doses. The volume of saline used as placebo will be identical to the challenge volume.

Hypertonic saline will be delivered via nebuliser during the induced sputum procedure according to the study-specific SOP.

9.4 Salbutamol

Volunteers undergoing the induced sputum procedure will be pre-treated with a prescribed dose of salbutamol (200mcg delivered via spacer) in accordance with the European Respiratory Society's guidelines [33].

9.5 MicroAIR NE-U22

This nebuliser is used for aerosol delivery of the BCG or placebo. This is an approved electromedical device, CE0197, EAN code 40 15672 10142 1. Information about the specifications, usage, and maintenance of the nebuliser device can be found in the device documentation (60). This is the same device used in studies TB026, TB035 and TB041 ((40), Clinicaltrials.gov NCT01954563 and NCT02709278).

9.6 Ultrasonic nebuliser NE-U780

This nebuliser is used for delivering the hypertonic saline during the induced sputum procedure. This is an approved electromedical device, CE0197, EN60601-1-2-2007. Information about the specifications, usage, and maintenance of the nebuliser device can be found in the device documentation [35].

9.7 Sedative & anaesthetic agents for bronchoscopy

Fentanyl is a licensed opioid used routinely to provide analgesia and sedation during medical procedures. It will be stored, dispensed and administered in accordance with standard NHS procedures and the SmPC.

Midazolam is a licensed benzodiazepine used routinely to provide sedation and amnesia during medical procedures. It will be stored, dispensed and administered in accordance with standard NHS procedures and the SmPC.

Lignocaine is a licensed local anaesthetic used routinely during medical procedures. It will be stored, dispensed and administered in accordance with standard NHS procedures and the SmPC.

Other licensed drugs may be used during bronchoscopy at the discretion of the Respiratory consultant performing the bronchoscopy and/or the Investigators.

10 ASSESSMENT OF SAFETY

Safety will be assessed by the frequency, incidence and nature of adverse events and serious adverse events arising during the study, as defined in Schedule of Visits and Procedures Section 3.2.

10.1 Definitions

Although the BCG challenge agent does not constitute an IMP, definitions for safety purposes are based on those used for IMPs.

Adverse Event (AE)

An AE is any untoward medical occurrence in a volunteer, which may occur during or after administration of BCG and does not necessarily have a causal relationship with the intervention. An AE can therefore be any unfavourable and unintended sign (including an abnormal laboratory finding), symptom or disease temporally associated with the study intervention, whether or not considered related to the study intervention.

Each adverse event will be graded by the participant according to the table for grading severity of adverse events (see Section 10.6). Severity gradings may be reviewed and discussed with the participants at the clinic visits.

Adverse Reaction (AR)

An AR is any untoward or unintended response to BCG. This means that a causal relationship between the agent and an AE is at least a reasonable possibility, i.e., the relationship cannot be ruled out. All cases judged by the reporting medical Investigator as having a reasonable suspected causal relationship to BCG (i.e. possibly, probably or definitely related to it) will qualify as adverse reactions.

Unexpected Adverse Reaction

An adverse reaction, the nature or severity of which is not consistent with the applicable product information (e.g. SmPC).

Serious Adverse Event (SAE)

An SAE is an AE that results in any of the following outcomes, whether or not considered related to the study intervention:

- Death.
- Life-threatening event (i.e., the volunteer was, in the view of the Investigator, at immediate risk of death from the event that occurred). This does not include an AE that, if it occurred in a more severe form, might have caused death.
- Persistent or significant disability or incapacity (i.e., substantial disruption of one's ability to carry out normal life functions).
- Hospitalisation or prolongation of existing hospitalisation, regardless of length of stay, even if it is a precautionary measure for continued observation. Hospitalisation (including inpatient or outpatient hospitalisation for an elective procedure) for a pre-existing condition that has not worsened unexpectedly does not constitute a serious AE.
- An important medical event (that may not cause death, be life threatening, or require hospitalisation) that may, based upon appropriate medical judgment, jeopardise the volunteer and/or require medical or surgical intervention to prevent one of the outcomes

listed above. Examples of such medical events include allergic reaction requiring intensive treatment in an emergency room or clinic, blood dyscrasias, or convulsions that do not result in inpatient hospitalisation.

- Congenital anomaly or birth defect.

Serious Adverse Reaction (SAR)

An adverse event (expected or unexpected) that is both serious and, in the opinion of the reporting Investigator or Sponsors, believed to be possibly, probably or definitely due to BCG or any other study treatments, based on the information provided.

Suspected Unexpected Serious Adverse Reaction (SUSAR)

A serious adverse reaction, the nature and severity of which is not consistent with the information about the medicinal product in question set out in the SmPC.

NB: To avoid confusion or misunderstanding the following note of clarification is provided: “Severe” is often used to describe intensity of a specific event, which may be of relatively minor medical significance. “Seriousness” is the regulatory definition supplied above.

10.2 Foreseeable Adverse Reactions

Foreseeable adverse reactions are listed in section 5.3.

10.3 Expected Serious Adverse Events

No serious adverse events are expected in this study.

10.4 Causality Assessment

For every AE, an assessment of the relationship of the event to the administration of BCG will be undertaken. An intervention-related AE refers to an AE for which there is a probable or definite relationship to administration of BCG. An interpretation of the causal relationship of the intervention to the AE in question will be made, based on the type of event, the relationship of the event to the time of BCG administration and the known biology of BCG, with reference to the SmPC (Table 4). Causality assessment will take place during any interim analyses and at the final safety analysis.

Table 4. Guidelines for assessing the relationship of challenge administration to an AE

0	**No Relationship**	No temporal relationship to BCG; ***and***<br>Alternate aetiology (clinical state, environment or other interventions); ***and***<br>Does not follow known pattern of response to BCG
1	**Unlikely**	Unlikely temporal relationship to BCG; ***and***<br>Alternate aetiology likely (clinical state, environment or other interventions); ***and***<br>Does not follow known typical or plausible pattern of response to BCG
2	**Possible**	Reasonable temporal relationship to BCG; ***or***<br>Event not readily produced by clinical state, environment or other interventions; ***or***<br>Similar pattern of response to that seen with other challenge agents
3	**Probable**	Reasonable temporal relationship to BCG; ***and***<br>Event not readily produced by clinical state, environment or other interventions; ***and***<br>Known pattern of response seen with other challenge agents
4	**Definite**	Reasonable temporal relationship to BCG; ***and***

		Event not readily produced by clinical state, environment or other interventions; ***and***<br>Known pattern of response seen with BCG

10.5 Reporting Procedures for All Adverse Events

All AEs occurring in the 14 days following challenge and the 7 days following bronchoscopy observed by the Investigator or reported by the volunteer, whether or not attributed to BCG, will be recorded on electronic diary cards. Data from the diary cards will be extracted following the last volunteer last visit (LVLV) or at any time prior to this in order to perform an interim safety analysis. Outside the diary card periods, respiratory and systemic AEs (listed in Table 5 below) will be specifically solicited at each visit, and graded by severity (as detailed in section 10.6). All AEs starting after the diary card period(s), or persisting after this period, will be recorded in the AE line listing of the CRF.

The overall appearance of the lung mucosa will be assessed at the bronchoscopy.

All AEs that result in a volunteer's withdrawal from the study will be followed up until a satisfactory resolution occurs, or until a non- study related causality is assigned (if the volunteer consents to this). Serious adverse events (SAEs) will be collected throughout the entire study period.

Table 5. Routinely solicited adverse events

	**Adverse event**
**Respiratory**	Cough
	Sore throat
	Tickly throat
	Wheeze
	Shortness of breath
	Coughing up phlegm
	Coughing up blood
	Chest tightness
	Chest pain
**Systemic**	Documented fever (oral temperature > 37.5° C)
	Myalgia
	Arthralgia
	Feverishness
	Headache
	Fatigue
	Nausea
	Malaise

Reporting Procedures for Serious AEs (see Safety Reporting SOP)

SAEs will be reported on the SAE forms to members of the study team immediately, once the Investigators become aware of their occurrence, as described in the study-specific SOP. Copies of all reports will be forwarded for review to the Chief Investigator (as the Sponsor's representatives) within 24 hours of the Investigator being aware of the suspected SAE. The Safety Monitoring Committee (SMC) will be notified of SAEs which are deemed possibly, probably or definitely related to study interventions; the SMC will be notified immediately (within 24 hours) of the Investigators' being aware of their occurrence. SAEs will not normally be reported immediately to the ethical committee(s) unless

there is a clinically important increase in occurrence rate, an unexpected outcome, or a new event that is likely to affect safety of study volunteers, at the discretion of the Chief Investigator and/or SMC.

Reporting Procedures for SARs
These will be reported as per any SAE.

Reporting Procedures for SUSARs
The Chief Investigator will report all SUSARs to the ethical committee(s) within required timelines (15 days for all SUSARs, unless life threatening in which case 7 days, with a final report within a further 8 days (total 15)). The Chief Investigator will also inform all Investigators concerned of relevant information about SUSARs that could adversely affect the safety of participants.

All SUSARs and deaths occurring during the study will be reported to the Sponsor. For all deaths, available autopsy reports and relevant medical reports will be made available for reporting to the relevant authorities.

Any serious adverse event considered by the CI to be related to the challenge agent and unexpected will be reported to the REC. As the challenge agent has a Marketing Authorisation, the mechanism for reporting any SAEs to the MHRA is via yellow card.

10.6 Assessment of Severity

The severity of clinical AEs will be assessed according to the scale in Tables 6 &7:

Table 6. Severity grading criteria for physical observations.

	**Grade 1 (mild)**	**Grade 2 (moderate)**	**Grade 3 (severe)**
Fever (oral)	37.6°C - 38.0°C	38.1°C – 39.0°C	>39.0°C
Tachycardia (bpm)*	101 - 115	116 – 130	>130
Bradycardia (bpm)**	50 – 54	40 – 49	<40
Systolic hypertension (mmHg)	141 - 159	160 – 179	≥180
Diastolic hypertension (mmHg)	91 - 99	100 – 109	≥110
Systolic hypotension (mmHg)***	85 - 89	80 – 84	<80

*Taken after ≥10 minutes at rest; **When resting heart rate is between 60 – 100 beats per minute. Use clinical judgement when characterising bradycardia among some healthy volunteer populations, for example, conditioned athletes; ***Only if symptomatic (e.g. dizzy/ light-headed)

Table 7. Severity grading criteria for respiratory and systemic AEs

**GRADE 0**	None
**GRADE 1**	Mild: Transient or mild discomfort (< 48 hours); no medical intervention/therapy required
**GRADE 2**	Moderate: Mild to moderate limitation in activity – some assistance may be needed; no or minimal medical intervention/therapy required
**GRADE 3**	Severe: Marked limitation in activity, some assistance usually required; medical intervention/therapy required, hospitalisation possible

10.7 Procedures to be followed in the event of abnormal findings

Eligibility for enrolment in the study in terms of laboratory findings will be assessed as detailed in Section 8.4. Abnormal clinical findings from medical history, examination or blood tests will be assessed as to their clinical significance throughout the study. Laboratory adverse events will be assessed using the TMF. If a test is deemed clinically significant, it may be repeated, to ensure it is not a single occurrence. If a test remains clinically significant, the volunteer will be informed and medical care arranged as appropriate and with the permission of the volunteer. Specific details regarding findings, discussion with volunteers and resulting actions will be recorded in the CRF. Decisions to exclude the volunteer from enrolling in the study or to withdraw a volunteer from the study will be at the discretion of the Investigator.

10.8 Safety Monitoring Committee

An independent Safety Monitoring Committee (SMC) will be appointed to provide real-time safety oversight. The SMC will be notified within 24 hours of the Investigators' being aware of the occurrence of SAEs. The SMC has the power to place the study on hold if deemed necessary following a study intervention-related SAE. The SMC will be chaired by Dr Hassan Mahomed with Professor Stephen Gorden and statistician Francesca Little as committee members. All correspondence between Investigator and SMC will be conveyed by the Investigator to the study Sponsor. The Chair of the SMC will be contacted for advice and independent review by the Investigator or study Sponsor in the following situations:

- Following any SAE deemed to be possibly, probably, or definitely related to BCG.
- Any other situation where the Investigator or study Sponsor feels independent advice or review is important.

Safety Profile Review

The safety profile will be assessed on an on-going basis by the Investigators. The Chief Investigator and relevant Investigators (as per the study delegation log) will also review safety issues and SAEs as they arise.

11 STATISTICS

Statistical support will be provided by an ongoing collaboration with the statistical team at the Nuffield Department of Primary Care Health Sciences Clinical trials Unit (safety and non-transcriptomic data) and with the Jenner Transcriptomic Core Facility (transcriptomic data).

This is an exploratory study with descriptive endpoints. Our previous experience with clinical studies suggests that this sample size is a feasible number to recruit, screen, enrol, and follow up in practical terms, whilst also allowing the determination of any substantial and clinically significant differences in safety and immunity between the BCG and control arms. Previous studies looking at differential gene expression (DGE) in vaccination studies have demonstrated significant levels of DGE in 1100 genes with 10-12 subjects/group (31). Studies using controlled human malaria infection have successfully identified a cellular immune response, CD38+CD4+ T cells with cytotoxic potential, which associates with protection in 7-10 subjects/group(61). In our intradermal BCG infection studies, we were able to show that mycobacterial recovery was inversely correlated with PPD-specific ELISpot responses using 12 subjects per group (37). Gene signatures which defined protective antibody responses to an influenza vaccine could be established using 24 subjects (62). In a similar study with a candidate HIV vaccine, 35 subjects in total were sufficient to define a gene signature of innate responses that predicted subsequent CD8+ T cell responses (63). We will evaluate 10 infected subjects at each of 5 time points (N=50) and, assuming control subject responses are negative, expect to pool data from the 3 saline controls at each point (N=15).

A detailed statistical analysis plan will be prepared prior to the data lock at the end of the study. Any deviations from this plan will be detailed in the analysis report.

Primary objective

The laboratory markers of innate and adaptive immunity will be summarised separately for the BCG and control arms using means and standard deviations. Log transformations will be considered where data are not normally distributed, and if this fails, the median and interquartile range will instead be presented. Statistical comparisons between groups will be made using t-tests or Mann-Whitney tests where appropriate, although it will be remembered that the study was not powered to detect significant statistical differences.

We will select key independent variables across all non-transcriptomic assays and combine into one model to determine if a combined model including both innate, cellular and humoral data is better able to predict control of mycobacterial growth than each outcome alone (64, 65).

To capture the dynamic transcriptional responses over time versus baseline, a functional principal component based analysis will be applied to all time points (66). We will identify the minimum number of transcripts required to accurately classify protected and non-protected individuals, using the PBMC MGIA data to define protection.

We will attempt in an exploratory analysis to combine the non-transcriptomic and transcriptomic datasets in order to improve the predictive value of our model.

Secondary objective

The secondary objective will be assessed by correlating different components of the innate immune response with the adaptive response using Spearman's rho. We will also correlate mycobacterial growth in PBMC at D56 with these innate and adaptive immune responses.

Tertiary objective

The tertiary objective, describing the human response to aerosol BCG challenge, will be assessed through collection of AEs as described in Section 3.2. The following parameters will be assessed:

- Occurrence of local signs and symptoms for 14 days following BCG (or placebo); for 7 days following bronchoscopy; and at each visit
- Occurrence of systemic signs and symptoms for 14 days following BCG (or placebo); for 7 days following bronchoscopy; and at each visit
- Bronchoscopy report
- Change from baseline for safety laboratory measures for 28 days following BCG (or placebo)
- Occurrence of serious adverse events during the whole study duration

This AE data will be tabulated and frequency, duration and severity of AEs compared between the two groups (BCG N=50: placebo N=15). Proportions in each group will be compared using the Chi-squared test or Fisher's exact test for small numbers. Continuous outcomes such as duration will be compared between groups using t-tests or Mann-Whitney tests where appropriate.

12 DATA MANAGEMENT

12.1 Source Data

Source documents are where data are first recorded, and from which participants' CRF data are obtained. These include, but are not limited to, the CRF itself (history and examination), the volunteer consent form, blood and microbiology results, radiology report, GP response letter, copy of bronchoscopy report and any further correspondence relating to the volunteer regarding medical/clinical issues. The CRF will be electronic and paper together with electronic diary cards.

CRF entries will be considered source data if the CRF is the site of the original recording (e.g. there is no other written or electronic record of data). All documents will be stored safely in confidential conditions. On all study-specific documents, other than the contact form, signed consent, the chest X-ray report, GP/medical correspondence and the bronchoscopy report, the participant will be referred to by the study participant number/code, not by name.

12.2 Access to Data

Direct access will be granted to authorised representatives from the Sponsor, host institution and the regulatory authorities to permit study-related monitoring, audits and inspections. All information relating to the study and its volunteers will be held in strict confidence, and in accordance with GCP and institutional requirements.

12.3 Data Recording and Record Keeping

The CI will be responsible for collecting, recording, analysing, and storing all the data accruing from the study. These tasks may be delegated to other Investigators. Paper CRFs will be stored in a key-locked cabinet at the CCVTM, and electronic CRFs on the OpenClinica™ or the e-diary ITCRC LTD databases, which are stored electronically on secure servers that are outsourced by OpenClinica™ or ITCRC respectively. Data from paper CRFs will be transcribed onto the OpenClinica™ database. Some data may be duplicated anonymously into an electronic Microsoft Excel™ file on the CCVTM secure server for clinical monitoring through the study.

Study records will be held by the Investigator for as long as required by anticipated legislation as a minimum (2 years) in order to enable dissemination of study results after publication and to enable decoding and destruction of anonymised samples if subsequently requested by a volunteer. Data will subsequently be transferred to a secure archive in accordance with the Data Protection Act. Data is archived for 25 years and then destroyed. This does not include the retention of bank details, which are destroyed following the completion of volunteer compensation. Volunteers will be assigned individual unique study numbers for identification on all study records, except where the use of identifiable information is unavoidable (including on GP correspondence, registration documents, and consent forms).

13 QUALITY ASSURANCE PROCEDURES

13.1 Quality Assurance

Investigator procedures

Approved SOPs will be used at all clinical and laboratory sites.

Modification to protocol

No substantial amendments to this protocol will be made without consultation with, and agreement of, the Sponsor. Any substantial amendments to the study that appear necessary during the course of the study must be discussed by the CI and Sponsor concurrently. If agreement is reached concerning

the need for an amendment, it will be produced in writing by the CI and will be made a formal part of the protocol following ethical approval.

An administrative change to the protocol is one that modifies administrative and logistical aspects of a protocol but does not affect the volunteers' safety, the scientific value of the study, the conduct of the study or safety of BCG. An administrative change is a non-substantial amendment and does not require REC approval. The CI is responsible for ensuring that changes to an approved study, during the period for which REC approval has already been given, are not initiated without REC review and approval except to eliminate apparent immediate hazards to the volunteer.

Protocol deviation

Any deviations from the protocol will be documented in a protocol deviation form and filed in the trial master file accordingly.

13.2 Monitoring

Data will be evaluated for compliance with the protocol and accuracy in relation to source documents. The study will be conducted in accordance with procedures identified in the protocol. Regular monitoring will be performed according to ICH GCP. According to applicable SOPs, the Monitors will verify that the clinical study is initiated, conducted and completed, and data are generated, documented and reported in compliance with the protocol, GCP and the applicable regulatory requirements.

14 SERIOUS BREACHES

A serious breach is defined as "A breach of GCP or the study protocol which is likely to affect to a significant degree:

- the safety or physical or mental integrity of the subjects of the study
- the scientific value of the study"

In the event that a serious breach is suspected, the Sponsor will contact the REC within 7 days within becoming aware of the breach of GCP.

15 ETHICAL AND REGULATORY CONSIDERATIONS

15.1 Declaration of Helsinki

The Investigator will ensure that this study is conducted in accordance with the principles of the Declaration of Helsinki as agreed by the World Medical Association General Assembly (Washington 2002).

15.2 Good Clinical Practice

The Investigator will ensure that this study is conducted in accordance with ICH Good Clinical Practice (GCP), and local regulatory requirements.

15.3 Approvals

A copy of the protocol, proposed informed consent form, other written volunteer information and the proposed advertising material will be submitted to an independent REC for written approval. The Investigators will submit and, where necessary, obtain approval from the REC for all subsequent substantial amendments to the protocol and informed consent document. The Investigators will notify

deviations from the protocol or SAEs occurring at the site to the Sponsor and will notify the REC of these in accordance with local procedures.

15.4 Reporting

The CI shall submit once a year throughout the study, or on request, an Annual Progress Report to the REC, host organisation and Sponsor. In addition, an End of Study notification and final report will be submitted to the REC, host organisation and Sponsor.

15.5 Volunteer Confidentiality

The study staff will ensure the volunteers' anonymity is maintained. All documents will be stored securely and only accessible by study staff and authorised personnel. No information concerning the study or the data will be released to any unauthorised third party, without prior written approval of the Sponsor. The study will comply with the Data Protection Act, which requires data to be anonymised as soon as it is practical to do so.

15.6 Expenses and Benefits

Volunteers will be compensated *pro rata* for their time, travel and for study procedures while participating in the study, amounting to a total of between £605 and £645 depending on the exact number of visits, and whether any repeat or additional visits are necessary.

16 FINANCE AND INSURANCE

16.1 Funding

This study will be financed by a research grant from The Wellcome Trust, held by Professor Helen McShane.

16.2 Indemnity

If any volunteer is harmed as a result of this study, medical care will be provided under the NHS.

Negligent Harm

Indemnity and/or compensation for negligent harm arising specifically from an accidental injury for which the University is legally liable as the Research Sponsor will be covered by the University of Oxford. NHS indemnity operates in respect to clinical treatment that is provided.

Non-Negligent Harm

Indemnity and/or compensation for harm arising specifically from an accidental injury, and occurring as a consequence of the Research volunteers' participation in the study for which the University is the Research Sponsor will be covered by the University of Oxford.

16.3 Insurance

The University has a specialist insurance policy in place - Newline Underwriting Management Ltd, at Lloyd's of London - which would operate in the event of any volunteer suffering harm as a result of their involvement in the research.

17 PUBLICATION POLICY

When the study is complete, a manuscript describing the primary study results will be written and published in a peer-reviewed, open access journal. International guidelines will be followed regarding authorship. There may also be secondary publications on more exploratory results.

18 REFERENCES

1. Dye C, Scheele S, Dolin P, Pathania V, Raviglione MC. Consensus statement. Global burden of tuberculosis: estimated incidence, prevalence, and mortality by country. WHO Global Surveillance and Monitoring Project. JAMA : the journal of the American Medical Association. 1999;282(7):677-86.
2. WHO. Global tuberculosis report 2017. Geneva http://www.who.int/tb/publications/global_report/en/ (Accessed 12 March 2018): World Health Organization; 2017. Contract No.: CC BY-NCSA 3.0 IGO.
3. Corbett EL, Marston B, Churchyard GJ, De Cock KM. Tuberculosis in sub-Saharan Africa: opportunities, challenges, and change in the era of antiretroviral treatment. Lancet (London, England). 2006;367(9514):926-37.
4. Colditz GA, Brewer TF, Berkey CS, Wilson ME, Burdick E, Fineberg HV, et al. Efficacy of BCG vaccine in the prevention of tuberculosis. Meta-analysis of the published literature. Jama. 1994;271(9):698-702.
5. Fine PE. The BCG story: lessons from the past and implications for the future. Rev Infect Dis. 1989;11 Suppl 2:S353-9.
6. Trunz BB, Fine P, Dye C. Effect of BCG vaccination on childhood tuberculous meningitis and miliary tuberculosis worldwide: a meta-analysis and assessment of cost-effectiveness. Lancet (London, England). 2006;367(9517):1173-80.
7. Rodrigues LC, Diwan VK, Wheeler JG. Protective effect of BCG against tuberculous meningitis and miliary tuberculosis: a meta-analysis. International journal of epidemiology. 1993;22(6):1154-8.
8. Tameris MD, Hatherill M, Landry BS, Scriba TJ, Snowden MA, Lockhart S, et al. Safety and efficacy of MVA85A, a new tuberculosis vaccine, in infants previously vaccinated with BCG: a randomised, placebo-controlled phase 2b trial. Lancet (London, England). 2013;381(9871):1021-8.
9. ~~Ndiaye BP, Thienemann F, Ota M, Landry BS, Camara M, Dieye S, et al. Safety, immunogenicity, and efficacy of the candidate tuberculosis vaccine MVA85A in healthy adults infected with HIV-1: a randomised, placebo-controlled, phase 2 trial. The Lancet Respiratory medicine. 2015;3(3):190-200.~~
10. ~~Scriba TJ, Coussens AK, Fletcher HA. Human Immunology of Tuberculosis. Microbiology spectrum. 2016;4(4).~~
11. ~~Brighenti S, Andersson J. Local immune responses in human tuberculosis: learning from the site of infection. The Journal of infectious diseases. 2012;205 Suppl 2:S316-24.~~
~~12. Flynn JL, Chan J, Triebold KJ, Dalton DK, Stewart TA, Bloom BR. An essential role for interferon gamma in resistance to Mycobacterium tuberculosis infection. J Exp Med. 1993;178(6):2249-54.~~
~~13. Newport MJ, Huxley CM, Huston S, Hawrylowicz CM, Oostra BA, Williamson R, et al. A mutation in the interferon-gamma-receptor gene and susceptibility to mycobacterial infection. The New England journal of medicine. 1996;335(26):1941-9.~~
14. ~~Flynn JL, Goldstein MM, Chan J, Triebold KJ, Pfeffer K, Lowenstein CJ, et al. Tumor necrosis factor-alpha is required in the protective immune response against Mycobacterium tuberculosis in mice. Immunity. 1995;2(6):561-72.~~
~~15. Keane J, Gershon S, Wise RP, Mirabile-Levens E, Kasznica J, Schwieterman WD, et al. Tuberculosis associated with infliximab, a tumor necrosis factor alpha-neutralizing agent. The New England journal of medicine. 2001;345(15):1098-104.~~
16. ~~Caruso AM, Serbina N, Klein E, Triebold K, Bloom BR, Flynn JL. Mice deficient in CD4 T cells have only transiently diminished levels of IFN-gamma, yet succumb to tuberculosis. Journal of immunology (Baltimore, Md : 1950). 1999;162(9):5407-16~~.
17. ~~Jones BE, Young SM, Antoniskis D, Davidson PT, Kramer F, Barnes PF. Relationship of the manifestations of tuberculosis to CD4 cell counts in patients with human immunodeficiency virus infection. Am Rev Respir Dis. 1993;148(5):1292-7.~~
18. ~~Behar SM, Dascher CC, Grusby MJ, Wang C-R, Brenner MB. Susceptibility of Mice Deficient in CD1D or TAP1 to Infection with Mycobacterium tuberculosis. The Journal of Experimental Medicine. 1999;189(12):1973-80.~~
19. ~~De Libero G, Singhal A, Lepore M, Mori L. Nonclassical T Cells and Their Antigens in Tuberculosis. Cold Spring Harbor Perspectives in Medicine. 2014;4(9):a018473.~~
20. ~~Khader SA, Bell GK, Pearl JE, Fountain JJ, Rangel-Moreno J, Cilley GE, et al. IL-23 and IL-17 in the establishment of protective pulmonary CD4+ T cell responses after vaccination and during Mycobacterium tuberculosis challenge. Nature immunology. 2007;8(4):369-77~~.
21. ~~Fletcher HA, Snowden MA, Landry B, Rida W, Satti I, Harris SA, et al. T-cell activation is an immune correlate of risk in BCG vaccinated infants. Nat Commun. 2016;7:11290.~~

22. Sia JK, Georgieva M, Rengarajan J. Innate Immune Defenses in Human Tuberculosis: An Overview of the Interactions between Mycobacterium tuberculosis and Innate Immune Cells. Journal of immunology research. 2015;2015:747543.
23. Morrison J, Pai M, Hopewell PC. Tuberculosis and latent tuberculosis infection in close contacts of people with pulmonary tuberculosis in low-income and middle-income countries: a systematic review and meta-analysis. The Lancet Infectious diseases. 2008;8(6):359-68.
24. Tian T, Woodworth J, Skold M, Behar SM. In vivo depletion of CD11c+ cells delays the CD4+ T cell response to Mycobacterium tuberculosis and exacerbates the outcome of infection. Journal of immunology (Baltimore, Md : 1950). 2005;175(5):3268-72.
25. Blomgran R, Ernst JD. Lung neutrophils facilitate activation of naive antigen-specific CD4+ T cells during Mycobacterium tuberculosis infection. Journal of immunology (Baltimore, Md : 1950). 2011;186(12):7110-9.
26. Esin S, Batoni G. Natural killer cells: a coherent model for their functional role in Mycobacterium tuberculosis infection. Journal of innate immunity. 2015;7(1):11-24.
27. Gold MC, Napier RJ, Lewinsohn DM. MR1-restricted mucosal associated invariant T (MAIT) cells in the immune response to Mycobacterium tuberculosis. Immunological reviews. 2015;264(1):154-66.
28. De Libero G, Mori L. The T-Cell Response to Lipid Antigens of Mycobacterium tuberculosis. Frontiers in immunology. 2014;5:219.
29. Shen Y, Zhou D, Qiu L, Lai X, Simon M, Shen L, et al. Adaptive immune response of Vgamma2Vdelta2+ T cells during mycobacterial infections. Science. 2002;295(5563):2255-8.
30. Mogues T, Goodrich ME, Ryan L, LaCourse R, North RJ. The relative importance of T cell subsets in immunity and immunopathology of airborne Mycobacterium tuberculosis infection in mice. J Exp Med. 2001;193(3):271-80.
31. Matsumiya M, Stylianou E, Griffiths K, Lang Z, Meyer J, Harris SA, et al. Roles for Treg expansion and HMGB1 signaling through the TLR1-2-6 axis in determining the magnitude of the antigen-specific immune response to MVA85A. PloS one. 2013;8(7):e67922.
32. Kleinnijenhuis J, Quintin J, Preijers F, Joosten LA, Ifrim DC, Saeed S, et al. Bacille Calmette-Guerin induces NOD2-dependent nonspecific protection from reinfection via epigenetic reprogramming of monocytes. Proceedings of the National Academy of Sciences of the United States of America. 2012;109(43):17537-42.
33. Urdahl KB. Understanding and overcoming the barriers to T cell-mediated immunity against tuberculosis. Seminars in immunology. 2014;26(6):578-87.
34. Behr MA, Wilson MA, Gill WP, Salamon H, Schoolnik GK, Rane S, et al. Comparative genomics of BCG vaccines by whole-genome DNA microarray. Science. 1999;284(5419):1520-3.
35. Marsay L, Matsumiya M, Tanner R, Poyntz H, Griffiths KL, Stylianou E, et al. Mycobacterial growth inhibition in murine splenocytes as a surrogate for protection against Mycobacterium tuberculosis (M. tb). Tuberculosis (Edinburgh, Scotland). 2013;93(5):551-7.
36. Fletcher HA, Tanner R, Wallis RS, Meyer J, Manjaly ZR, Harris S, et al. Inhibition of mycobacterial growth in vitro following primary but not secondary vaccination with Mycobacterium bovis BCG. Clinical and vaccine immunology : CVI. 2013;20(11):1683-9.
37. Harris SA, Meyer J, Satti I, Marsay L, Poulton ID, Tanner R, et al. Evaluation of a human BCG challenge model to assess antimycobacterial immunity induced by BCG and a candidate tuberculosis vaccine, MVA85A, alone and in combination. The Journal of infectious diseases. 2014;209(8):1259-68.
38. Randall TD, Mebius RE. The development and function of mucosal lymphoid tissues: a balancing act with micro-organisms. Mucosal immunology. 2014;7(3):455-66.
39. Zhou QT, Leung SS, Tang P, Parumasivam T, Loh ZH, Chan HK. Inhaled formulations and pulmonary drug delivery systems for respiratory infections. Advanced drug delivery reviews. 2015;85:83-99.
40. Rau JL. The inhalation of drugs: advantages and problems. Respiratory care. 2005;50(3):367-82.
41. Cusumano CL, Jernigan JA, Waldman RH. Aerosolized BCG (Tice strain) treatment of bronchogenic carcinoma: phase I study. J Natl Cancer Inst. 1975;55(2):275-9.
42. Garner FB, Meyer CA, White DS, Lipton A. Aerosol BCG treatment of carcinoma metastatic to the lung: a phase I study. Cancer. 1975;35(4):1088-94.
43. Satti I, Meyer J, Harris SA, Manjaly Thomas ZR, Griffiths K, Antrobus RD, et al. Safety and immunogenicity of a candidate tuberculosis vaccine MVA85A delivered by aerosol in BCG-vaccinated healthy adults: a phase 1, double-blind, randomised controlled trial. The Lancet Infectious diseases. 2014;14(10):939-46.

44. WHO. IVR strategic plan 2010 - 2020. http://whqlibdoc.who.int/hq/2010/WHO_IVB_10.02_eng.pdf?ua=1 (accessed 02 March 2015) 2010.
45. Rosenthal SR, McEnery JT, Raisys N. Aerogenic BCG vaccination against tuberculosis in animal and human subjects. J Asthma Res. 1968;5(4):309-23.
46. ~~Nuermberger EL, Yoshimatsu T, Tyagi S, Bishai WR, Grosset JH. Paucibacillary tuberculosis in mice after prior aerosol immunization with Mycobacterium bovis BCG. Infect Immun. 2004;72(2):1065-71.~~
47. ~~White AD, Sarfas C, West K, Sibley LS, Wareham AS, Clark S, et al. Evaluation of the Immunogenicity of Mycobacterium bovis BCG Delivered by Aerosol to the Lungs of Macaques. Clinical and vaccine immunology : CVI. 2015;22(9):992-1003.~~
48. ~~England PH. Unlicensed BCG Vaccine (Freeze Dried) supplied by InterVax Ltd, Canada and manufactured by BB NCIPD Ltd, Bulgaria. 2017.~~
49. ~~Santosuosso M, McCormick S, Roediger E, Zhang X, Zganiacz A, Lichty BD, et al. Mucosal luminal manipulation of T cell geography switches on protective efficacy by otherwise ineffective parenteral genetic immunization. Journal of immunology (Baltimore, Md : 1950). 2007;178(4):2387-95.~~
50. White AD, Sibley L, Dennis MJ, Gooch K, Betts G, Edwards N, et al. Evaluation of the safety and immunogenicity of a candidate tuberculosis vaccine, MVA85A, delivered by aerosol to the lungs of macaques. Clinical and vaccine immunology : CVI. 2013;20(5):663-72.
51. ~~Sakala IG, Kjer-Nielsen L, Eickhoff CS, Wang X, Blazevic A, Liu L, et al. Functional heterogeneity and anti-mycobacterial effects of mouse mucosal associated invariant T (MAIT) cells specific for riboflavin metabolites(). Journal of immunology (Baltimore, Md : 1950). 2015;195(2):587-601.~~
52. ~~Sakai S, Kauffman KD, Schenkel JM, McBerry CC, Mayer-Barber KD, Masopust D, et al. Cutting edge: control of Mycobacterium tuberculosis infection by a subset of lung parenchyma-homing CD4 T cells. Journal of immunology (Baltimore, Md : 1950). 2014;192(7):2965-9.~~
53. ~~Du Rand IA, Blaikley J, Booton R, Chaudhuri N, Gupta V, Khalid S, et al. British Thoracic Society guideline for diagnostic flexible bronchoscopy in adults: accredited by NICE. Thorax. 2013;68(Suppl 1):i1-i44.~~
54. ~~Bernasconi M, Koegelenberg CFN, Koutsokera A, Ogna A, Casutt A, Nicod L, et al. Iatrogenic bleeding during flexible bronchoscopy: risk factors, prophylactic measures and management. ERJ Open Research. 2017;3(2):00084-2016.~~
55. ~~England PH. Unlicensed BCG Vaccine (Freeze Dried) supplied by InterVax Ltd, Canada and manufactured by BB NCIPD Lts, Bulgaria. 2018.~~
56. ~~Ritz N, Tebruegge M, Connell TG, Sievers A, Robins-Browne R, Curtis N. Susceptibility of Mycobacterium bovis BCG Vaccine Strains to Antituberculous Antibiotics. Antimicrobial Agents and Chemotherapy. 2009;53(1):316-8.~~
57. ~~Kariyawasam HH, Aizen M, Kay AB, Robinson DS. Safety and tolerability of three consecutive bronchoscopies after allergen challenge in volunteers with mild asthma. Thorax. 2007;62(6):557-8.~~
58. ~~Elston WJ, Whittaker AJ, Khan LN, Flood-Page P, Ramsay C, Jeffery PK, et al. Safety of research bronchoscopy, biopsy and bronchoalveolar lavage in asthma. Eur Respir J. 2004;24(3):375-7.~~
59. ~~Busse WW, Wanner A, Adams K, Reynolds HY, Castro M, Chowdhury B, et al. Investigative bronchoprovocation and bronchoscopy in airway diseases. American journal of respiratory and critical care medicine. 2005;172(7):807-16.~~
60. ~~OMRON. OMRON Microair U22 website http://www.omron-healthcare.com/eu/en/our-products/respiratory-therapy/microair-u22 (accessed 12 March 2018). 201~~8.
61. ~~Burel JG, Apte SH, Groves PL, Klein K, McCarthy JS, Doolan DL. Reduced Plasmodium Parasite Burden Associates with CD38+ CD4+ T Cells Displaying Cytolytic Potential and Impaired IFN-γ Production. PLoS pathogens. 2016;12(9):e1005839.~~
62. ~~Tan Y, Tamayo P, Nakaya H, Pulendran B, Mesirov JP, Haining WN. Gene signatures related to B-cell proliferation predict influenza vaccine-induced antibody response. European journal of immunology. 2014;44(1):285-95.~~
63. ~~Zak DE, Andersen-Nissen E, Peterson ER, Sato A, Hamilton MK, Borgerding J, et al. Merck Ad5/HIV induces broad innate immune activation that predicts CD8(+) T-cell responses but is attenuated by preexisting Ad5 immunity. Proceedings of the National Academy of Sciences of the United States of America. 2012;109(50):E3503-12.~~
64. Liquet B, Cao K-AL, Hocini H, Thiébaut R. A novel approach for biomarker selection and the integration of repeated measures experiments from two assays. BMC Bioinformatics. 2012;13(1):325.
65. Mooney M, McWeeney S, Canderan G, Sekaly RP. A systems framework for vaccine design. Current opinion in immunology. 2013;25(5):551-5.

66. Wu S, Wu H. More powerful significant testing for time course gene expression data using functional principal component analysis approaches. BMC Bioinformatics. 2013;14(1):6.

19 Appendices: Appendix A

a.

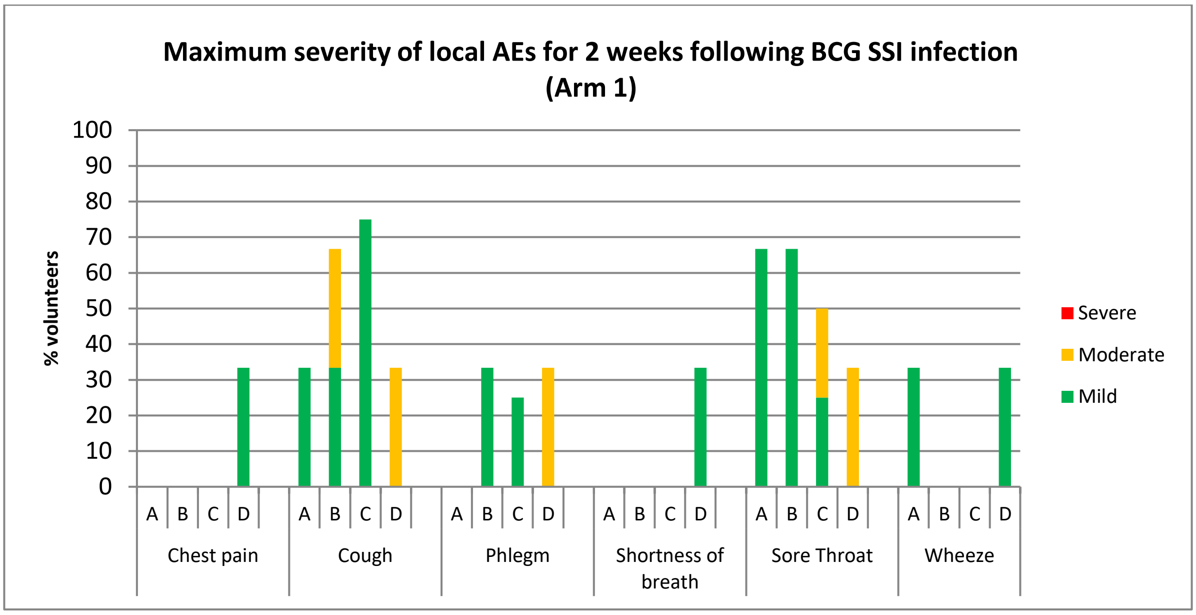

**Note no haemoptysis reported for any volunteer*

b.

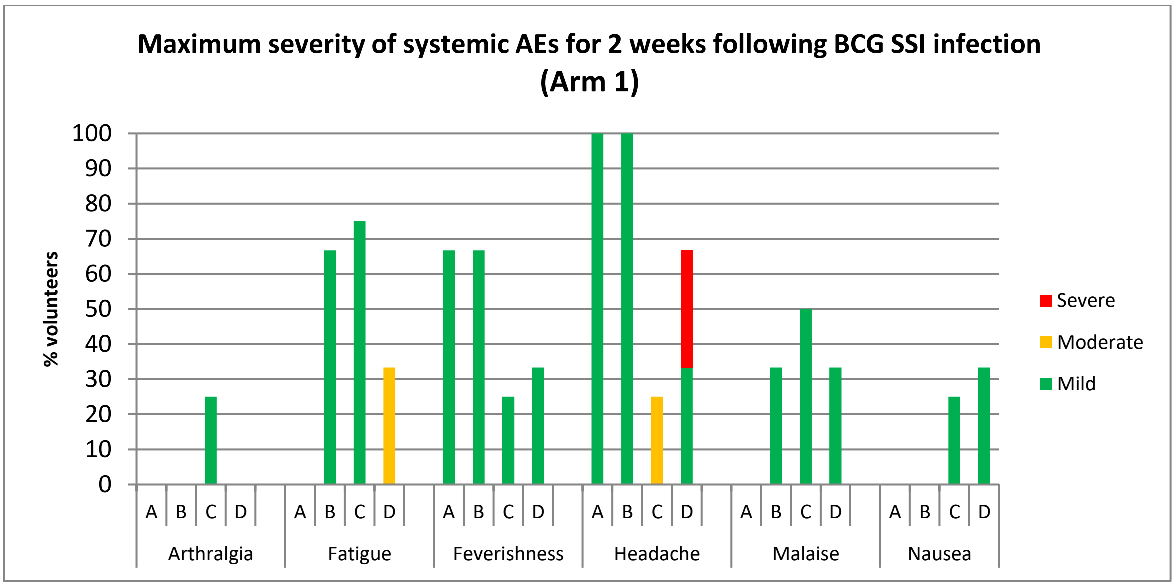

**Note no myalgia or fever (T>37.5) reported for any volunteer*

Figure 1: *(**a**) Local and (**b**) systemic solicited adverse events for the 2 weeks following BCG SSI challenge as reported in volunteer diary card and at follow up visits by Group (1A-1D). Group 1A (n=3): 1 x 10^3 BCG SSI by aerosol; Group 1B (n=3): 1 x 10^4 BCG SSI by aerosol; Group 1C (n=4): 1 x 10^5 BCG SSI by aerosol; Group 1D (n=3): 1 x 10^5 BCG SSI by intradermal injection*

a.

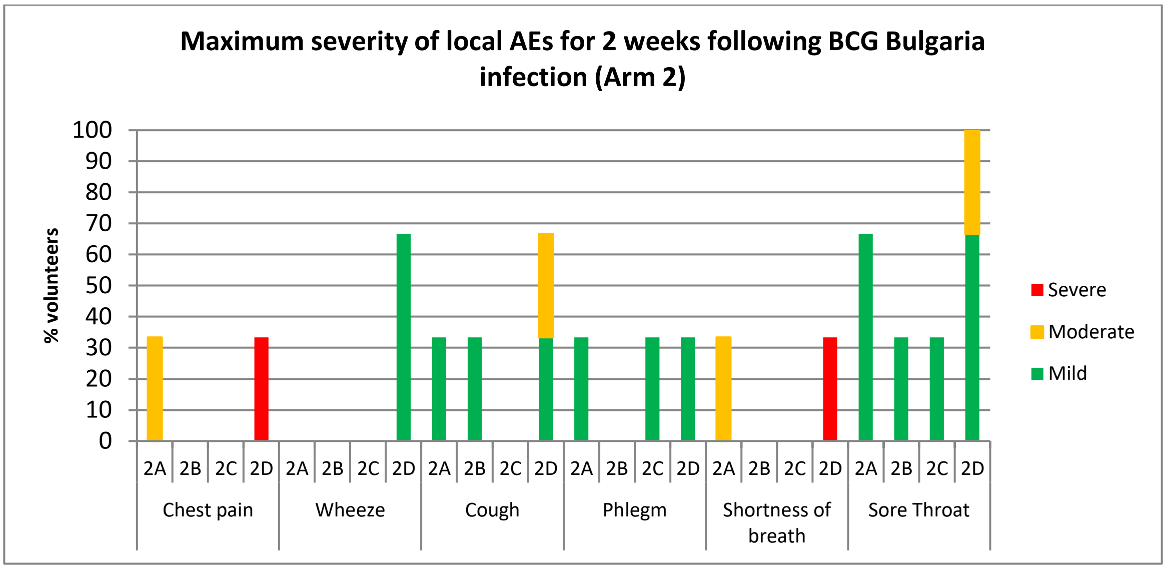

**Note no haemoptysis reported for any volunteer*

b.

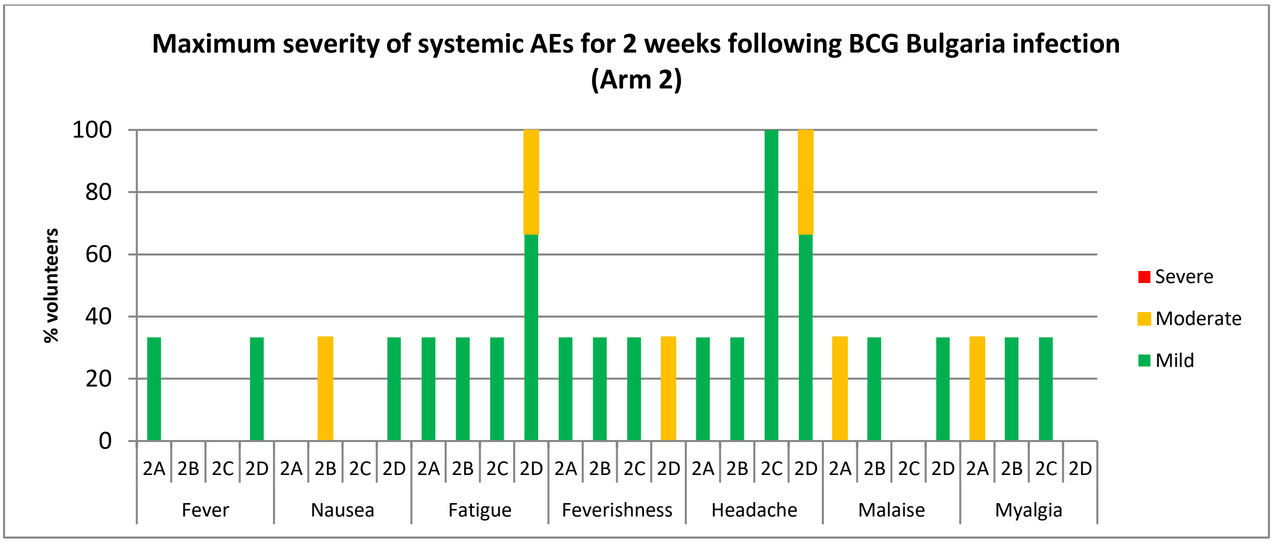

Figure 2: *(**a**) Local and (**b**) systemic solicited adverse events for the 2 weeks following BCG Bulgaria challenge in Arm 2 as reported in volunteer diary card and at follow up visits by Group (2A-2D). Group 2A (n=3): 1 x 10^4 BCG Bulgaria by aerosol; Group 2B (n=3): 1 x 10^5 BCG Bulgaria by aerosol; Group 2C (n=3): 1 x 10^6 BCG Bulgaria by aerosol; Group 2D (n=3): 1 x 10^7 BCG Bulgaria by aerosol*

Table 1: Combined unsolicited AEs and laboratory AEs in 2 week period following BCG challenge Arms 1 and 2

Unsolicited events for 2 weeks after receiving BCG SSI challenge via aerosol (Groups 1A-1C) or intradermally (Group 1D) or after receiving BCG Bulgaria via aerosol (Groups 2A-2D) which are of special interest (respiratory tract symptoms) or which were considered possibly, probably or definitely related to vaccination

Group	Subject ID	Unsolicited symptoms and laboratory AEs	Time-point	Duration	Grading	Relatedness
1A	TBT-0411001	Flushed face	Day 1	2 hours	1	Possibly
1A	TBT-0411002	Light headedness	Day 0	30 mins	1	Possibly
1B	TBT-0411005	Cold symptoms - Rhinorrhoea and sneezing	Day 9-10	2 days	1	Unlikely
1C<br>1C	TBT-0411011<br>TBT-0411011	Tickle at back of throat<br>Blocked nose	Day 0<br>Day 1	1 day<br>2 days	1<br>1	Probably<br>Possibly
1C	TBT-0411008	Stuffy nose	Day 1; 2; 6 - 10	6 days total	1	Possibly
1D<br>1D<br>1D	TBT-0415501<br>TBT-0415501<br>TBT-0415501	Bilateral ear pain<br>Blocked nose with resultant 2 week upper respiratory tract infection (URTI) with cough and fatigue as recorded under solicited AEs<br>Elevated CRP with concurrent URTI.	Day 0<br>Day 2<br>Day 7	3 days<br>3 days<br>1 week	1<br>1<br>1	Unlikely<br>Unlikely<br>Unlikely
2A	TBT-0411017	Elevated CRP (noted Day 2 -no baseline- peaked at 29.1mg/L day 14, resolved on repeat at Day 28) with concurrent inflamed toe (?gout) followed by discogenic cervical pain requiring co-codamol, ibuprofen and physio	Day 2	2- 4 weeks	N/A	Possibly
2A	TBT-0411019	Blocked nose/congestion	Day 9	2 days	2	Possibly
2A	TBT-0411019	Dyspnoea and pleuritic right sided chest pain on background of viral URTI. A+E attendance on advise of 111 hence graded 2 but otherwise volunteer would have graded pain and dyspnoea 1. Diagnosed in A+E as pleurisy secondary to viral infection.	Day 15	1 day	2	Possibly
2B	TBT-0411024	Chest irritation<br>Chest irritation with associated dry cough on using spirometer	Day 0<br>Day 9	3 days<br>2 days	1<br>1	Possibly<br>Possibly
2C	TBT-0411025	Slightly runny nose	Day 1	1 day	1	Possibly
2C	TBT-0411029	Slightly runny nose	Day 2	2 days	1	Possibly
2C	TBT-0411025	Lymphocytopaenia (pre-existing Grade 1, fell to Grade 2 following BCG with trough at Day 28 prior to normalising)	Day 0	32 days	2	Possibly
2D	TBT-0411033	Diarrhoea (one episode loose stool)	Day 1	Once	1	Possibly
		Cold symptoms/runny nose.<br>Associated with general malaise, chest pain and SOB	Day 8	5 days	2	Possibly
		Dizziness	Day 9	2 days	2	Possibly
		Tachycardia	Day 9	2 days	2	Possibly
		Loss of consciousness	Day 10	Few minutes	3	Possibly
		Chest tightness	Day 10	2 days	2	Possibly
		Laryngitis (“lost voice”)	Day 13	2 days	2	Possibly
2D	TBT-0415503	Runny nose	Day 0	2 days	1	Possibly
2D	TBT-0415504	Blocked sinus	Day 1	3 days	2	Unlikely
		Epistaxis	Day 10	Once	1	Unlikely

Table 2: Unsolicited AEs and laboratory AEs for 6 month period following BCG infection

Group	Subject ID	Unsolicited symptoms and laboratory AEs	Time-point	Duration	Grading	Relatedness
1B	TBT-0411003	Left lower lobe pneumonia post bronchoscopy. Mycobacterial sputum culture negative. Treated with oral broad spectrum antibiotics for 7 days with full resolution.	Day 14	5 days	2	Unlikely
1B	TBT-0411003	Elevated white cell count and CRP consistent with concurrent pneumonia	Day 14	7 days	1	Unlikely
1B	TBT-0411005	Rhinorrhoea, malaise	Day 79	5 days	1	Unlikely
1B	TBT-0411006	Fever to 38.2<br>Severe headache resulting in one day off work<br>Cough productive of sputum. Could not exercise at the gym	Day 145<br><br>Day 147	1 days<br>3 days<br>8 days	2<br>3<br>1	Unlikely<br>Unlikely<br>Unlikely
1C	TBT-0411007	Sore throat<br>Tickly cough<br>Coughing up phlegm in the morning	Day 25	7 days	1	Unlikely
1C<br>1C	TBT-0411011<br>TBT-0411011	Tickle at back of throat<br>Headache<br>Nausea<br>Blocked nose	Day 26<br>Day 77	5 days<br>3 days	1<br>1	Unlikely<br>Unlikely
1C	TBT-0411015	Runny nose<br>Itchy eyes<br>Cough<br>Fatigue	Day 18-20	3	1	Unlikely<br>Unlikely<br>Unlikely<br>Unlikely
2A	TBT-0411017	Ring of itchy blisters left wrist. Not painful. Likely reaction to spider bite. To consider possibly atypical shingles.	5 months	2 weeks	1	Unlikely
2A	TBT-0411017	Dry cough<br>Sore throat	6 months	4 days	1	Unlikely
2B	TBT-0411021	URTI (cough, sore throat, fatigue)	Day 26	3 days	1	Unlikely
2B	TBT-0411023	Sore throat<br>Cough productive of yellow phlegm	3 months	2 days	1	Unlikely
2B	TBT-0411024	URTI (sinus pain, runny nose, malaise, fatigue, feverishness, afebrile)	2 months	4 days	1	Unlikely
2C	TBT-0411025	Sore throat	3 months	5 days	1	Unlikely
2C	TBT-0411029	Dry tickly cough since bronchoscopy	D14	2 weeks	1	Possibly
2C	TBT-0411031	Dry cough<br>Sore throat	D21	1 week	1	Possibly
