# Supplementary material for: Early mucosal responses following a randomised controlled human inhaled infection with attenuated Mycobacterium bovis BCG
Source: Nat Commun. 2025 May 29;16:4989. doi: 10.1038/s41467-025-60285-4 (PMC12122720; doi:10.1038/s41467-025-60285-4)
Supplement: Supplementary file 2 — Description Of Additional Supplementary File [file 41467_2025_60285_MOESM2_ESM.pdf]

Description of Additional supplementary files

Supplementary Data 1:

Gene sets enriched among upregulated genes in pDCs from BCG-vaccinated volunteers compared to saline controls on day 7. Enriched gene sets were those with adjusted p values < 0.05.
