# Supplementary material for: Early mucosal responses following a randomised controlled human inhaled infection with attenuated Mycobacterium bovis BCG
Source: Nat Commun. 2025 May 29;16:4989. doi: 10.1038/s41467-025-60285-4 (PMC12122720; doi:10.1038/s41467-025-60285-4)
Supplement: Supplementary file 4 — Reporting summary [file 41467_2025_60285_MOESM4_ESM.pdf]

nature portfolio

Corresponding author(s): Helen McShane

Last updated by author(s): 28/04/25

Reporting Summary

Nature Portfolio wishes to improve the reproducibility of the work that we publish. This form provides structure for consistency and transparency in reporting. For further information on Nature Portfolio policies, see our Editorial Policies and the Editorial Policy Checklist.

Statistics

For all statistical analyses, confirm that the following items are present in the figure legend, table legend, main text, or Methods section.

n/a	Confirmed	
☒	☐	The exact sample size (n) for each experimental group/condition, given as a discrete number and unit of measurement
☐	☒	A statement on whether measurements were taken from distinct samples or whether the same sample was measured repeatedly
☐	☒	The statistical test(s) used AND whether they are one- or two-sided<br>*Only common tests should be described solely by name; describe more complex techniques in the Methods section.*
☐	☒	A description of all covariates tested
☐	☒	A description of any assumptions or corrections, such as tests of normality and adjustment for multiple comparisons
☐	☒	A full description of the statistical parameters including central tendency (e.g. means) or other basic estimates (e.g. regression coefficient) AND variation (e.g. standard deviation) or associated estimates of uncertainty (e.g. confidence intervals)
☐	☒	For null hypothesis testing, the test statistic (e.g. F, t, r) with confidence intervals, effect sizes, degrees of freedom and P value noted<br>*Give P values as exact values whenever suitable.*
☒	☐	For Bayesian analysis, information on the choice of priors and Markov chain Monte Carlo settings
☒	☐	For hierarchical and complex designs, identification of the appropriate level for tests and full reporting of outcomes
☐	☒	Estimates of effect sizes (e.g. Cohen's d, Pearson's r), indicating how they were calculated

Our web collection on statistics for biologists contains articles on many of the points above.

Software and code

Policy information about availability of computer code

Data collection	Gen5 (v2.07)<br>AID (v7.0 iSpot)<br>ediary ITCRC (v2.0.1 - 15239e7)<br>OpenClinica Enterprise (v3 and v4)<br>BD FACSDiva 8.0.2

Data analysis

FlowJo (BD) v10.
ndp.view2 software (v2.8.24).
R version 4.1.2
Python 3.8.19
GraphPad Prism (v9 and 10)
Cell Ranger Software Suite (v.6.0.1)
SoupX (v1.6.1)
Scanpy (v1.9.8)
Scrublet (v0.2.3)
harmonypy (v0.0.6)
leidenalg (v0.8.10)
umap-learn (v0.5.3)
DESeq2 (v1.34.0)
clusterProfiler (v4.2.2)
SPSS v.25

For manuscripts utilizing custom algorithms or software that are central to the research but not yet described in published literature, software must be made available to editors and reviewers. We strongly encourage code deposition in a community repository (e.g. GitHub). See the Nature Portfolio guidelines for submitting code & software for further information.

Data

Policy information about availability of data

All manuscripts must include a data availability statement. This statement should provide the following information, where applicable:

- Accession codes, unique identifiers, or web links for publicly available datasets
- A description of any restrictions on data availability
- For clinical datasets or third party data, please ensure that the statement adheres to our policy

scRNA data has been deposited at GSE282132
The gene signature for T1-T17 Subpop 1 and 2 is available at https://ars.els-cdn.com/content/image/1-s2.0-S1074761322001753-mmc5.xlsx
The gene signature for PPD response is available at Table S8 of https://www.science.org/doi/10.1126/sciadv.adq8229#supplementary-materials
For all other data, any reasonable request for raw or analysed data will be reviewed by the study team, and a response can be expected within 14 days. The data generated in this study are subject to patient confidentiality, and the transfer of data or materials will require approval from the sponsor. Any shared data will be de-identified. Requests should be made to HMcS. Source data are provided with this paper.

Research involving human participants, their data, or biological material

Policy information about studies with human participants or human data. See also policy information about sex, gender (identity/presentation), and sexual orientation and race, ethnicity and racism.

Reporting on sex and gender

Biological sex data was collected and self-reported. Gender identity data was not collected. Volunteers were assessed for eligibility and allocated to groups without consideration of biological sex. Post-enrolment analysis of biological sex allocation to groups were performed and are provided in Extended Data 1B. 80% (8/10) BCG D2 volunteers, 70% (7/10) BCG D7 volunteers, 100% (3/3) D2 saline volunteers and 33% (1/3) D7 saline volunteers were female (biological sex). Other data is not disaggregated by sex due to small sample size. Consent was not obtained for this breakdown – and given the small numbers in each group, such a breakdown could result in inadvertent identification of volunteers

Reporting on race, ethnicity, or other socially relevant groupings

Country of birth and ethnicity were collected. This was self identified information and the wording was recorded as nominated by the volunteer. See Extended data 1B. Confounding was addressed through randomisation of volunteers.

Population characteristics

Demographic characteristics are provided in Extended data 1B.

Recruitment

Healthy UK adults aged 18-50 residing in Oxford were recruited by use of approved advertisements. Following written informed consent, volunteers were screened for eligibility. Volunteers who fulfilled the inclusion criteria were invited to participate.

Ethics oversight

The study protocol and all documents were approved by the South Central Oxford A REC on 20 September 2018 (18/SC/0307). The study was registered with clinicaltrials.gov prior to study commencement (NCT03912207, April 11, 2019) and was conducted at the Oxford University Hospitals NHS Trust and the Centre for Clinical Vaccinology and Tropical Medicine, University of Oxford, according to the principles of the Declaration of Helsinki and Good Clinical Practice.

Note that full information on the approval of the study protocol must also be provided in the manuscript.

Field-specific reporting

Please select the one below that is the best fit for your research. If you are not sure, read the appropriate sections before making your selection.

☒ Life sciences ☐ Behavioural & social sciences ☐ Ecological, evolutionary & environmental sciences

Life sciences study design

All studies must disclose on these points even when the disclosure is negative.

Sample size	The sample size of 10 per group was determined on the basis of our previous experience with phase 1 exploratory clinical studies and chosen with the aim of detecting substantial differences in the primary outcome measure between the two groups, while also being a feasible number to enrol. The sample size was not determined with the aim of achieving statistical significance
Data exclusions	Statistical analysis was performed on the completed randomised controlled groups. Blood from volunteer TBT-04301029 D56 (D7 BCG volunteer) was one day of out window but was included in the analysis as this was perceived as unlikely to significantly impact results given the window range was 28 days. Not all analyses were performed on all volunteers due to restrictions with sample volume as outlined in the manuscript. This was expected and built into the study design analysis with innate flow cytometry panels prioritised over more experimental adaptive panels to ensure primary outcomes met.<br>For the antibody analysis, there were three missing BAL samples due to processing errors.
Replication	Due to limited sample availability, there was no attempt to replicate, except for ELISpot analysis.
Randomization	Volunteers were sequentially enrolled by the study investigator into either Group 1 (n=13) or Group 2 (n=13) and randomised 10:3 to inhale either BCG or saline by variable randomisation using sequentially numbered sealed envelopes, prepared by an independent statistician.
Blinding	Volunteers and the bronchoscopist reporting on the appearance of the airway were blinded to the inhaled agent.

Reporting for specific materials, systems and methods

We require information from authors about some types of materials, experimental systems and methods used in many studies. Here, indicate whether each material, system or method listed is relevant to your study. If you are not sure if a list item applies to your research, read the appropriate section before selecting a response.

Materials & experimental systems

n/a	Involved in the study	
☐	☒	Antibodies
☒	☐	Eukaryotic cell lines
☒	☐	Palaeontology and archaeology
☒	☐	Animals and other organisms
☐	☒	Clinical data
☒	☐	Dual use research of concern
☒	☐	Plants

Methods

n/a	Involved in the study	
☒	☐	ChIP-seq
☐	☒	Flow cytometry
☒	☐	MRI-based neuroimaging

Antibodies

Antibodies used

CD103 PE Biolegend 350206
CD11c BV785 Biolegend 301644
CD14 BV421 ebioscience 48-0149-42
CD14 PE dazzle Biolegend 325634
CD14 PerCpCy5.5 Biolegend 301824
CD153 AF647 R&D Systems FAB1028R-100
CD16 AF488 Biolegend 302019
CD16 APC-Cy7 Biolegend 360710
CD161 BV785 Biolegend 339930
CD19 ECD Biolegend 302252
CD19 BV510 BD Biosciences 562947
CD206 APC-CY7 Biolegend 321120
CD26 PE Biolegend 302706
CD27 BV605 Biolegend 302830
CD29 APC-Cy7 Biolegend 303014
CD3 BUV496 BD Biosciences 564809
CD3 AF700 ebioscience 56-0038-42
CD326 PercpCy5.5 Biolegend 369804
CD4 Pacific Blue Biolegend 300521
CD4 BUV395 BD Biosciences 564724
CD4 Pacific Blue Biolegend 300521
CD45 BV650 Biolegend 304044
CD49D BV605 Biolegend 304324

CD56 PECY5 Invitrogen 15-0567-42
CD56 BV785 Biolegend 362550
CD66b AF700 Biolegend 305114
CD86 APC Biolegend 305412
CD8a APC-H7 BD Biosciences 641400
CD8a BV650 Biolegend 301042
CXCR3 PerCpCy5.5 Biolegend 353714
HLADR BV650 Biolegend 307650
HLA-DR PE Biolegend 307606
IFN-γ PE-CY7 Life Technologies 25-7319-82
IL-17 AF488 Biolegend 512308
IL-2 PE Beckman coulter PNIM2718U
KLRG-1 AF488 Biolegend 367714
Live/Dead Aqua Invitrogen L34957
Live/Dead Red Invitrogen L23102
MR1 CTR: 6-FP AF488 NIH Tetramer Core facility n/a
MR1 TEM: 5-OP-RU APC NIH Tetramer Core facility n/a
PD-1 BUV395 BD Biosciences 745619
Siglec8 PE-Cy7 Biolegend 347112
TNF-α AF647 Biolegend 502916
Vα24 PercpCy5.5 Biolegend 360004
Vα7.2 BV421 Biolegend 351716
Vβ 11 PE-vio770 Miltenyi 130-108-734
γδ pan: B1 (11F2) BV711 BD Biosciences 745505
γδ2: (B6) BV480 BD Biosciences 746567

Validation

CD103 PE Biolegend 350206 https://www.biolegend.com/nl-be/products/pe-anti-human-cd103-integrin-alphae-antibody-6918
CD11c BV785 Biolegend 301644 https://www.biolegend.com/fr-fr/products/brilliant-violet-785-anti-human-cd11c-antibody-11920
CD14 BV421 ebioscience 48-0149-42 https://www.thermofisher.com/antibody/product/CD14-Antibody-clone-61D3-Monoclonal/404-0149-42
CD14 PE dazzle Biolegend 325634 https://www.biolegend.com/fr-ch/products/pe-dazzle-594-anti-human-cd14-antibody-9785
CD14 PerCpCy5.5 Biolegend 301824 https://www.biolegend.com/nl-be/products/percp-cyanine5-5-anti-human-cd14-antibody-4223?GroupID=BLG4805
CD153 AF647 R&D Systems FAB1028R-100 https://www.rndsystems.com/products/human-cd30-ligand-tnfsf8-alexa-fluor-647-conjugated-antibody-116614_fab1028r
CD16 AF488 Biolegend 302019 https://www.biolegend.com/en-ie/products/alexa-fluor-488-anti-human-cd16-antibody-2734
CD16 APC-Cy7 Biolegend 360710 https://www.biolegend.com/en-gb/products/apc-cyanine7-anti-human-cd16-antibody-9134?GroupID=BLG12171
CD161 BV785 Biolegend 339930 https://www.biolegend.com/ja-jp/products/brilliant-violet-785-anti-human-cd161-antibody-9973
CD19 ECD Biolegend 302252 https://www.biolegend.com/fr-ch/products/pe-dazzle-594-anti-human-cd19-antibody-9783?GroupID=BLG5913
CD19 BV510 BD Biosciences 562947 https://www.bdbiosciences.com/en-us/products/reagents/flow-cytometry-reagents/research-reagents/single-color-antibodies-ruo/bv510-mouse-anti-human-cd19.562947
CD206 APC-CY7 Biolegend 321120 https://www.biolegend.com/en-gb/products/apc-cyanine7-anti-human-cd206-mmr-antibody-5670?GroupID=BLG8520
CD26 PE Biolegend 302706 https://www.biolegend.com/en-ie/products/pe-anti-human-cd26-antibody-611
CD27 BV605 Biolegend 302830 https://www.biolegend.com/fr-ch/products/brilliant-violet-605-anti-human-cd27-antibody-7804?GroupID=BLG10174
CD29 APC-Cy7 Biolegend 303014 https://www.biolegend.com/fr-lu/products/apc-cyanine7-anti-human-cd29-antibody-3185
CD3 BUV496 BD Biosciences 564809 https://www.bdbiosciences.com/en-au/products/reagents/flow-cytometry-reagents/research-reagents/single-color-antibodies-ruo/buv496-mouse-anti-human-cd3.612940
CD3 AF700 ebioscience 56-0038-42 https://www.thermofisher.com/antibody/product/CD3-Antibody-clone-UCHT1-Monoclonal/56-0038-42
CD326 PercpCy5.5 Biolegend 369804 https://www.biolegend.com/en-ie/products/percp-cyanine5-5-anti-human-cd326-epcam-antibody-14154
CD4 Pacific Blue Biolegend 300521 https://www.biolegend.com/ja-jp/products/pacific-blue-anti-human-cd4-antibody-2850
CD4 BUV395 BD Biosciences 564724 https://www.bdbiosciences.com/en-au/products/reagents/flow-cytometry-reagents/research-reagents/single-color-antibodies-ruo/buv395-mouse-anti-human-cd4.564724
CD4 Pacific Blue Biolegend 300521 https://www.biolegend.com/ja-jp/products/pacific-blue-anti-human-cd4-antibody-2850
CD45 BV650 Biolegend 304044 https://www.biolegend.com/fr-lu/products/brilliant-violet-650-anti-human-cd45-antibody-8883
CD49D BV605 Biolegend 304324 https://www.biolegend.com/ja-jp/products/brilliant-violet-605-anti-human-cd49d-antibody-12838
CD56 PECY5 Invitrogen 15-0567-42 https://www.thermofisher.com/antibody/product/CD56-NCAM-Antibody-clone-CMSSB-Monoclonal/15-0567-42
CD56 BV785 Biolegend 362550 https://www.biolegend.com/fr-lu/products/brilliant-violet-785-anti-human-cd56-ncam-antibody-12129
CD66b AF700 Biolegend 305114 https://www.biolegend.com/en-ie/products/alexa-fluor-700-anti-human-cd66b-antibody-12098
CD86 APC Biolegend 305412
CD8a APC-H7 BD Biosciences 641400 https://www.biolegend.com/ja-jp/products/apc-anti-human-cd86-antibody-2864
CD8a BV650 Biolegend 301042 https://www.biolegend.com/nl-nl/products/brilliant-violet-650-anti-human-cd8a-antibody-7652
CXCR3 PerCpCy5.5 Biolegend 353714 https://www.biolegend.com/en-gb/products/percp-cyanine5-5-anti-human-cd183-cxcr3-antibody-7583
HLADR BV650 Biolegend 307650 https://www.biolegend.com/de-de/products/brilliant-violet-650-anti-human-hla-dr-antibody-8875
HLA-DR PE Biolegend 307606 https://www.biolegend.com/fr-ch/products/pe-anti-human-hla-dr-antibody-790
IFN-γ PE-CY7 Life Technologies 25-7319-82 https://www.thermofisher.com/antibody/product/IFN-gamma-Antibody-clone-4S-B3-

Monoclonal/25-7319-82
IL-17 AF488 Biolegend 512308 https://www.biolegend.com/fr-ch/products/alexa-fluor-488-anti-human-il-17a-antibody-4453?GroupID=BLG5454
IL-2 PE Beckman coulter PNIM2718U https://www.beckman.com.au/reagents/coulter-flow-cytometry/antibodies-and-kits/single-color-antibodies/il-2
KLRG-1 AF488 Biolegend 367714 https://www.biolegend.com/en-gb/products/alexa-fluor-488-anti-human-klrg1-mafa-antibody-21352
Live/Dead Aqua Invitrogen L34957 https://www.thermofisher.com/order/catalog/product/L34957
Live/Dead Red Invitrogen L23102 https://www.thermofisher.com/order/catalog/product/L23102
MR1 CTR: 6-FP AF488 NIH Tetramer Core facility https://tetramer.yerkes.emory.edu/reagents/mr1/4104
MR1 TEM: 5-OP-RU APC NIH Tetramer Core facility https://tetramer.yerkes.emory.edu/reagents/mr1/4103
PD-1 BUV395 BD Biosciences 745619 https://www.bdbiosciences.com/en-au/products/reagents/flow-cytometry-reagents/research-reagents/single-color-antibodies-ruo/buv395-mouse-anti-human-cd279-pd-1.745619
Siglec8 PE-Cy7 Biolegend 347112 https://www.biolegend.com/fr-ch/products/pe-cyanine7-anti-human-siglec-8-antibody-13602
TNF-α AF647 Biolegend 502916 https://www.biolegend.com/en-ie/products/alexa-fluor-647-anti-human-tnf-alpha-antibody-2751
Vα24 PercpCy5.5 Biolegend 360004 https://www.biolegend.com/en-gb/products/percpcyanine5-5-anti-human-tcr-valpha24-antibody-8868?GroupID=BLG11984
Vα7.2 BV421 Biolegend 351716 https://www.biolegend.com/fr-ch/products/brilliant-violet-421-anti-human-tcr-valpha7-2-antibody-8878
Vβ 11 PE-vio770 Miltenyi 130-108-734 https://www.miltenyibiotec.com/US-en/products/tcr-vb11-antibody-anti-human-reafinity-rea559.html
γδ pan: B1 (11F2) BV711 BD Biosciences 745505 https://www.bdbiosciences.com/en-sg/products/reagents/flow-cytometry-reagents/research-reagents/single-color-antibodies-ruo/bv711-mouse-anti-human-tcr.745505
γδ2: (B6) BV480 BD Biosciences 746567 https://www.bdbiosciences.com/en-au/products/reagents/flow-cytometry-reagents/research-reagents/single-color-antibodies-ruo/bv480-mouse-anti-human-v-2-tcr.746567

Clinical data

Policy information about clinical studies
All manuscripts should comply with the ICMJE guidelines for publication of clinical research and a completed CONSORT checklist must be included with all submissions.

Clinical trial registration

clinicaltrials.gov (NCT03912207)

Study protocol

Study protocol is provided

Data collection

Volunteers were enrolled at Centre for Clinical Vaccinology and Tropical Medicine (CCVTM), Oxford University. Enrolment commenced 19th March 2019 and completion for Group 1 and 2 was 30th July 2020 May 9, 2019. Volunteers received one dose of either aerosol 1x10^7 CFU BCG Danish or aerosol saline.
Safety was assessed by collecting frequency and severity of solicited and unsolicited adverse events (AEs) throughout the 6 month study period. Expected respiratory AEs (cough, sore throat, tickly throat, wheeze, dyspnoea, sputum production, haemoptysis, chest pain, chest tightness) and systemic AEs (fever, feverishness, fatigue, malaise, headache, myalgia, arthralgia, nausea) were solicited from subjects using an electronic diary card for 14 days post-infection (twice daily for the first 2 days then once daily in the evening), and reviewed at each clinic visit. Volunteers were supplied with a digital thermometer.
Blood biochemical and haematological parameters were measured on baseline and D28 post infection. Vital signs were measured at all clinic visits, and spirometry was measured at visits to 2 months post-infection unless clinically indicated. The transfer capacity of uptake of carbon monoxide (TLCO) was measured at baseline and at D7 post-infection.
Fibreoptic bronchoscopy was performed on volunteers at either 2 days (D2, Group 1) or 7 days (D7, Group 2) post-infection. using a standardised clinical protocol. Airway macroscopic appearance was reported and photographed. Bronchoalveolar lavage (BAL) was then collected from the right middle lobe (medial segment) using 100ml of 0.9% sodium chloride and up to six endobronchial biopsies of the subcarinal region were then taken, as tolerated. Induced sputum was collected at 3 and 6 months.
Blood was collected at every scheduled visit for exploratory immunology (Day 0 (D0), D2, D7, D14, D28, D56, D84, D168).

Outcomes

The primary outcome was to define the early systemic and mucosal innate and adaptive immune responses induced following aerosol BCG infection. Secondary outcome measures were to identify laboratory markers of the immune response that correlated with protection as defined by a peripheral blood mononuclear cell (PBMC) Mycobacterial Growth Inhibition Assay (MGIA). Tertiary outcomes were to describe the human clinical response to aerosol BCG challenge.

Plants

Seed stocks	N/A
Novel plant genotypes	N/A
Authentication	N/A

Flow Cytometry

Plots

Confirm that:

- [ ] The axis labels state the marker and fluorochrome used (e.g. CD4-FITC).
- [x] The axis scales are clearly visible. Include numbers along axes only for bottom left plot of group (a 'group' is an analysis of identical markers).
- [x] All plots are contour plots with outliers or pseudocolor plots.
- [x] A numerical value for number of cells or percentage (with statistics) is provided.

Methodology

Sample preparation	Fresh WB was stimulated at baseline (D0), D14, D28 and D56, and at either D2 (Group1) or D7 (Group 2) [53]. BAL cells were resuspended to achieve a concentration of 1x106 live lymphocytes/ml. 1ml BAL or WB were stimulated overnight with BCG Pasteur (1.2x106 CFU/ml; Aeras).<br>Four separate flow cytometry panels were used to detect cell types and antigen specific cytokine production (Extended Data 8). BAL cells were allocated sequentially starting at Panel 1. The number of panels used for each volunteer was limited by the amount of BAL cells available. Blood samples were stained with all four panels at baseline, D14, D28 and D56, and D2 (Group 1) or D7 (Group 2).<br>For phenotype staining, cells were stained for viability with Live/Dead stain (with the exception of lysed fixed WB cells) followed by surface staining. For panels analysing antigen presenting cells, cells were incubated with Fc-receptor blocker (Biolegend) alone prior to staining with antibody mix combined with Fc blocker. MAIT cells were detected by incubating with tetramers (MR1 5-OP-RU (MR1 MHC class I-related protein 1 5-OP-RU5-(2-oxopropylideneamino)-6-D-ribitylaminouracil); MR1 control 6-FP (6-formylpterin))) prior to surface staining for other markers. For detection of intracellular cytokine responses, after surface staining, cells were permeabilised and stained intracellularly.<br>All stained cells were fixed with 1% paraformaldehyde, acquired within 24 hours on an LSR Fortessa v.2 Std X20 flow cytometer using BD FACSDiva 8.0.2 and analysed on FlowJo (BD) v10.
Instrument	LSR Fortessa v.2 Std X20 flow cytometer
Software	Data collection was done using BD FACSDiva 8.0.2<br>Analysis was done using FlowJo (BD) v10.
Cell population abundance	No sample sorting for flow cytomtery was done.
Gating strategy	See Extended Data 8 for information on gating strategy for the four panels used

- [x] Tick this box to confirm that a figure exemplifying the gating strategy is provided in the Supplementary Information.
